# Approaches to Surgical Debridement in Necrotizing Soft Tissue Infections: Outcomes of an Animated, Interactive Survey

**DOI:** 10.1007/s00268-022-06470-8

**Published:** 2022-02-20

**Authors:** Jaco Suijker, Fabienne A. C. Hofmans, Paul P. M. van Zuijlen, Huib A. Cense, H. Jaap Bonjer, Annebeth Meij-de Vries

**Affiliations:** 1 Burn Center, Red Cross Hospital, Vondellaan 13, 1942 LE Beverwijk, The Netherlands; 2 Department of Plastic, Reconstructive and Hand Surgery, Amsterdam Movement Sciences Amsterdam UMC, location VUmc, Amsterdam, Netherlands; 3 Association of Dutch Burn Centers, Beverwijk, The Netherlands; 4 Pediatric Surgical Center, Emma Children′s Hospital, Amsterdam UMC, location AMC, Amsterdam, Netherlands; 5 Department of Surgery, Red Cross Hospital, Beverwijk, The Netherlands; 6 Department of Surgery, Amsterdam University Medical Center, Amsterdam, The Netherlands

## Abstract

**Background:**

Necrotizing soft tissue infections (NSTI) affect long-term quality of life in survivors. Different approaches to debridement may influence quality of life. The aim of this study was to assess the current practice of the debridement of NSTI in the Netherlands.

**Methods:**

An animated, interactive online survey was distributed among general surgeons and plastic surgeons in the Netherlands. Two NSTI-cases were presented, followed by questions regarding the preferred surgical approach. Case one described a woman with a swollen, red leg, with signs of sepsis and without visible necrosis. Case two described an immunocompromised man with septic shock syndrome and extensive necrosis.

**Results:**

In total 232 responses were included (143 general surgeons, 89 plastic surgeons). In case one, 32% chose to preserve all skin, while 17% chose to resect all skin above the affected fascia, including normal-looking skin. In case two, all participants resected necrotic skin, and most (88%) also blue discolored skin. While 32% did not resect more than blue discolored and necrotic skin, 35% also resected red-colored skin, and 21% all skin overlying the affected fascia, including normal colored skin. Respondents working in a hospital with a burn center tended to preserve more skin, whereas plastic surgeons chose more often for skin resection compared to general surgeons.

**Conclusions:**

By using a novel approach to a survey, the authors demonstrate the existence of extensive practice variety regarding the approach to debridement of NSTI among Dutch general and plastic surgeons. Consensus is needed, followed by targeted education of surgeons.

**Supplementary Information:**

The online version contains supplementary material available at 10.1007/s00268-022-06470-8.

## Introduction

Necrotizing soft-tissue infections (NSTIs) are severe, bacterial infections, characterized by rapid tissue destruction and systemic toxicity [1]. It includes subtypes based on tissue layers (necrotizing fasciitis, necrotizing myositis, necrotizing cellulitis) and based on anatomic region (Fournier gangrene, Ludwig’s angina) [2–4]. Due to improved disease management, the mortality rate has been decreasing from 28.5% on average in the late nineteenth century, to 19.7% more recently [5, 6]. As a result, more focus on long-term effects of this disease is needed. It is expected that the previously described decreased Health Related Quality of Life (HRQoL) in survivors of NSTI is at least partially explained by scars and scar-related issues (pain or itch, contractures, esthetical concerns) [7–10]. Therefore, limiting scar size and improving scar quality are expected to improve long-term outcomes.

One factor that may influence final scar size could be the approach used for the surgical debridement of NSTI in the acute phase. Historically *en bloc* debridement has been used, in which all skin above the affected fascia is resected, providing effective source control but leading to extensive skin defects*.* More recently, a skin-sparing approach to debridement was proposed, in which all potentially viable skin above the affected fascia is preserved, in order to decrease final scar size and scar-related problems (contractures, pain, appearance) [11, 12]. Although there is currently no convincing evidence for the superiority of either of these approaches, the skin-sparing approach has been adapted as the preferred approach in the Dutch guideline on NSTI [13, 14]. This guideline advises to ‘only remove non-vital skin and preserve all vital skin,’ to ‘preserve skin when its vitality is unsure,’ and to use a skin-sparing approach as described by Tom et al. [12] This has likely made most Dutch general surgeons and plastic surgeons, both proponents and opponents, aware of this novel skin-sparing approach.

This study was initiated to explore the current practice regarding the acute debridement of NSTI among general surgeons and plastic surgeons in the Netherlands, including characteristics related to, and motivations for the selected approach. To this end, an innovative approach (interactive, animated survey) was used, in order to more realistically mimicking the perioperative situation. In this original communication, the findings obtained in this study are described and discussed.

## Material and methods

Reporting of this survey is based on the ‘Checklist for Reporting Results of Internet E-surveys’ (CHERRIES). [15] Since no patients were involved, there was no need for ethical approval and no institutional review board was involved.

### Survey development

**Fig. 1:**
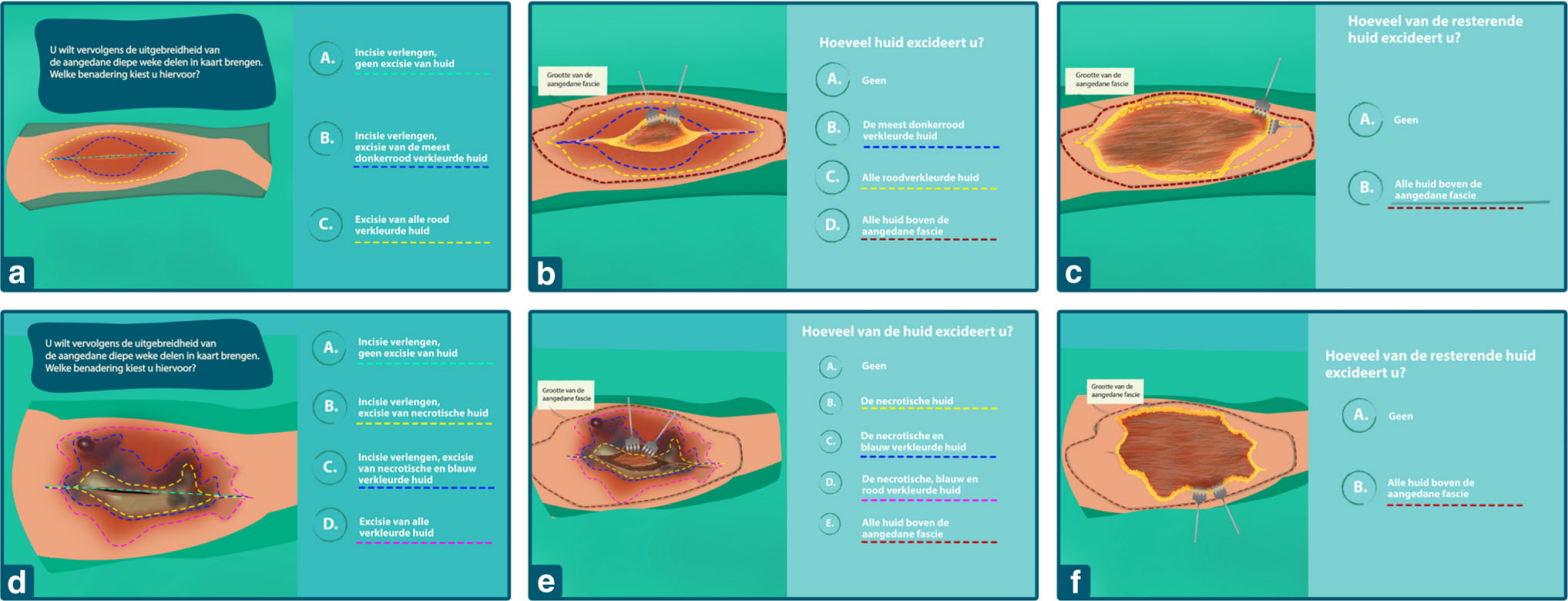
Slides from the survey demonstrating the course of the questions provided to participants in the two cases, with the different dotted lines indicating different incision patterns. In case one, the first question (**a**) had three answer possibilities. Depending on the answer provided, the appropriate follow-up question was selected. For example, when answer A was selected the follow-up question was as visible in (**b**), while when answer C was selected this follow-up question was as visible in (**c**). This was similar for case two, with the first question displayed in (**d**), followed by (**e**) if A was selected, and (**f**) if D was selected. For all possible slides, see Supplementary material, Appendix A

An animated, interactive survey was designed by a professional animator (Ruby Horstman Creative & Art Direction) in collaboration with an online host (Fresh TV Videomarketing). It was made in the Dutch language and based on two cases of patients with NSTI. First baseline characteristics of respondents were assessed, regarding type of surgical specialty, type of hospital employed, years of experience as a surgeon, and number of patients with NSTI treated (Table 1). All non-academic hospitals were classified as general hospital and further classified into general hospital with or without a burn center. Then, two case descriptions were provided to respondents (Table 2), each containing two image-based questions regarding the surgical approach of preference for each of the cases (Fig. 1). A link was made available to view the survey. (https://bit.ly/2PntknR) The course of the whole survey, including translation to the English language, can be viewed in supplementary material, Appendix A, slides 1–35.

**Table 1:** Questions regarding baseline characteristics and the distribution of these characteristics in the sample

Type of surgeon?	A plastic surgeon	89 (38%)
	B general surgeon	143 (62%)
Type of hospital?	A academic hospital without burns center	42 (18%)
	B general hospital with a burn center	29 (13%)
	C general hospital without a burn center	161 (69%)
Years of experience?	A 1–5 years	70 (30%)
	B 6–15 years	91 (39%)
	C > 15 years	71 (31%)
Number of patients with NSTI operated?	A < 10 patients	115 (50%)
	B ≥ 10 patients	117 (50%)

*NSTI* necrotizing soft-tissue infection

**Table 2:** Descriptions of the two presented cases in the survey

	Case one	Case two
Patient characteristics	40-year-old female	50-year-old male
Medical history	No relevant medical history	Rheumatoid arthritis for which he takes prednisone
Background	Has progressive pain since one day in her right calf, which started after exercising. The lower limb is progressively red and swollen since the morning. She slept poorly, has no appetite, vomited once and had a temperature of 39 degrees Celsius at home	Admitted yesterday to cardiology dpt. After a collapse with a small hematoma, pain on the left hip and persisting sinus tachycardia. Discoloration of the right hip widely expanded
Vital parameters	115 bpm	150 bpm
	110/70 mmHg	80/40 mmHg
		35.6 degrees Celsius
Upon presentation	Red, painful, swollen lower limb without evident necrosis	Unresponsive man with sepsis eci
*Small hematoma and pain left hip*		
Laboratory results and additional diagnostics	CRP 160 mg/L	CRP 410 mg/L
	Leukocytes 27 × 10^9/L	Leukocytes 2 × 10^9/L
		Lactate 5.0 mmol/L
		X-ray chest no abnormalities
		urine sediment clean

*bpm* beats per minute, *mmHg* millimeter of mercury, *mg/L* milligram/liter, *eci e causa ignota*; *CRP* C-reactive protein

### Sample recruitment

The survey was distributed among Dutch general surgeons and plastic surgeons by means of relevant associations (Dutch Association for Surgery and Dutch Association for Plastic Surgery). By means of this approach, all Dutch plastic surgeons (n = 359) were reached. Due to limitations in reaching general surgeons (no direct mailing of the invitation was possible, only adding a link to the website in the monthly newsletter), general surgeons were consequently approached by emails and WhatsApp invitations. This had also implications for the calculation of a response rate for a part of the surgeons, see supplementary material, Appendix B for more details. Recruitment started on the 11th of September 2020, and the survey closed on the 19th of November 2020, after which it remained accessible for anyone to view. After the survey closed, one additional question was sent to those respondents who voluntarily filled in their e-mail address. This question assessed underlying arguments for the chosen debridement approach in case one. (Supplementary material, Appendix A, slides 37–39) A one-time reminder was sent after one week.

### Data analysis

Surveys with completed questions regarding the characteristics and surgical approach were included for analysis. Surveys with non-interpretable data were excluded, which was the case if more than one answer option was selected. Descriptive data were presented as counts and percentages. To identify correlation between cases, as well as identify factors related to skin preservation during debridement, answers to the final questions of each case were transposed to an ordinal scale, and Spearman’s Rs and ordinal logistic regression were used, respectively. See Supplementary material, Appendix B for more details on transposing data and the analysis performed. Statistical significance was set at alpha < 0.05.

## Results

In total 243 general surgeons and plastic surgeons participated. The exact known response rate was 26% for participating plastic surgeons and 29% for a part of the participating general surgeons (those contacted by the newsletter). The estimated response rate for all general surgeons was 24%, which would suggest a combined total response rate of 25% for the whole sample.

After applying the inclusion criteria, 232 surveys were included for analysis, of which 143 (62%) respondents self-identified as general surgeon, 89 (38%) as plastic surgeon. Most respondents (n = 190, 82%) indicated working in a general hospital, which included a burn center in 29 (13%). More than 15 years of work experience was reported by 71 (31%) respondents, six to 15 years by 91 (39%), and one to five years by 70 (30%). Half (n = 117) reported to have treated ten or more patients with NSTI, the other half (n = 115) less than ten (Table 1).

### Case one

**Fig. 2:**
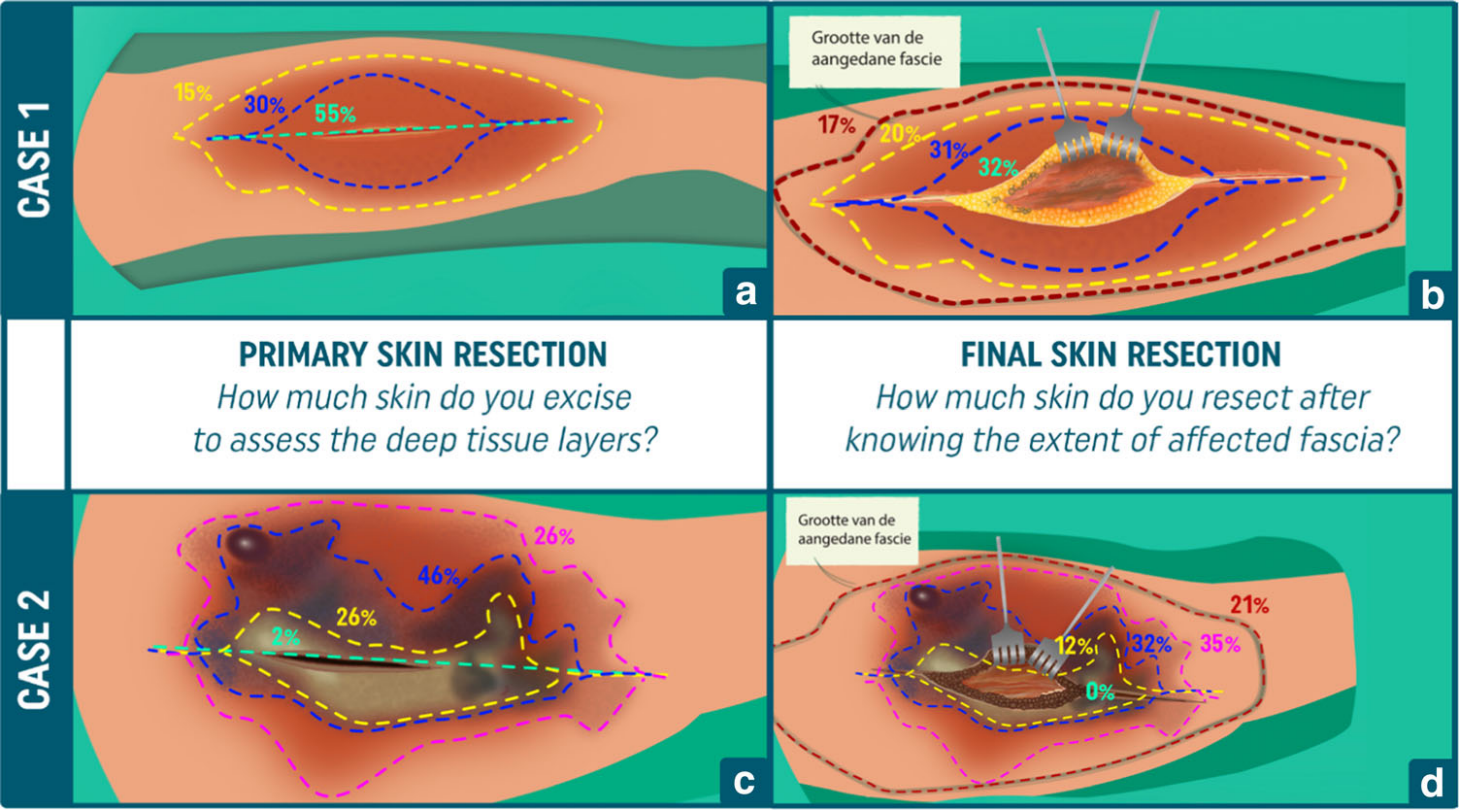
Distribution of the approaches chosen for the first and second question for both case one and case two. The different dotted lines indicate different possible incision patterns, and gray continuous line (visible in the second questions) the extent of involved deep tissue layers which were debrided. The percentages, which color math the relevant dotted lines, indicate how many percent of the respondents chose these different incision patterns. Responses to the first questions for case one and case two are displayed in a and c, and to final incision patterns in b and d, respectively

In response to the first question, assessing the approach to exploration of deep tissue layers, 129 (55%) respondents chose not to excise any skin. Seventy-one (30%) indicated to resect a part of the red skin, while 35 (15%) respondents chose to resect all erythematous skin (Fig. 2a).

After exploration of the deep tissue layers, the following final preferences were observed: 76 (32%) respondents did not resect any skin, 72 (31%) of the respondents chose a partial resection of red skin, 48 (20%) respondents resected all red skin, but no normal-looking skin, and 39 (17%) chose to resect all skin over the diseased fascia including not discolored, normal looking skin (Fig. 2b).

### Case two

The first question concerned the amount of skin (necrotic, blue discolored or red) excised to assess the extent of fascial involvement. Of the respondents, four (2%) chose to not excise any skin yet. Sixty-one (26%) indicated to resect only the necrotic skin before assessing the deep tissue layers, 108 (46%) chose to resect necrotic and blue discolored skin, while 62 (26%) respondents chose to resect all abnormal skin, including the red skin (Fig. 2c).

After final debridement, none would preserve necrotic skin, 27 (12%) preserved blue discolored skin, while the majority (88%) resected at least necrotic and blue discolored skin. While 32% did not resect more than blue discolored and necrotic skin, 35% would adjacently resect red skin (but preserve normal colored skin), and 21% would resect all skin overlying the affected fascia, including normal skin (Fig. 2d).

### Case comparison

A strong correlation was found between the approach chosen in case one and two (P < 0.001) with Spearman’s Rs 0.75. Of those who chose to preserve all skin in case 1, most (70%) chose to resect blue discolored skin in case 2.

### Factors related to surgical approach

Multivariate ordinal logistic regression analysis revealed two factors significantly related to the approach to skin resection. Employment in a general hospital with burn center was associated with increased preservation of skin (case two (1.131, SD 0.384–1.879, *P* = 0.003)) compared to those employed in a general hospital without a burn center. Plastic surgeons tended to resect more skin (case one (-0.518, SD -1.009–0.028, *P* = 0.038)/case two (– 0.830, SD – 1.335–0.326, *P* = 0.001)) in comparison to general surgeons. For an overview of all results, see Table 3.

**Table 3:** Results from the multivariate ordinal regression analysis on factors associated with a more (positive coefficient) or less (negative coefficient) skin preserving approach, for cases one and two

Variables	Regression coefficient	95% CI	*P* value
*Case one*			
Plastic surgeon	– 0.518	– 1.009, – 0.028	0.038
General hospital with BC	0.725	– 0.018, 1.469	0.056
Academic hospital	– 0.017	– 0.641, 0.607	0.958
6– 15 years of work experience	0.056	– 0.529, 0.641	0.851
> 15 years of work experience	0.204	– 0.463, 0.872	0.548
≥ 10 patients	0.371	– 0.159, 0.901	0.170
*Case two*			
Plastic surgeon	– 0.830	– 1.335, – 0.326	0.001
General hospital with BC	1.131	0.384, 1.879	0.003
Academic hospital	– 0.325	– 0.962, 0.313	0.318
6– 15 years of work experience	– 0.085	– 0.676, 0.507	0.779
> 15 years of work experience	– 0.287	– 0.963, 0.389	0.406
≥ 10 patients	0.283	– 0.253, 0.819	0.301

*CI* confidence interval, *BC* Burn center

### Motivation for approach

In total 126 respondents answered the additional question about the reason to resect or preserve skin. This resulted in a total of 152 given answers, due to the possibility to select multiple answers per question (Supplementary material, Appendix A, slides 37–39).

The main reason (85%) to preserve red skin overlying the affected fascia, which is characteristic of a skin-sparing approach, was to reduce scar size. (Table 4) The responders who excised (a part of or all) red skin, so not using a skin-sparing approach, mostly reasoned that the red skin eventually would become necrotic (41%). (Table 5) This was also argued most by responders who also excised normal-looking skin overlying the affected fascia (classic en bloc), alongside with the arguments that they learned it this way (33%) and resection of this normal-looking skin would decrease bacterial load (33%). (Table 5).

**Table 4:** Underlying arguments provided for the preservation of red-colored skin

	Preserved red skin (N = 47)
Excision of the red skin does not contribute to a decrease in disease progression	10 (21%)
This skin might stay vital, and because of that can lead to less extensive scars	40 (85%)
It is recommended like this in the Dutch guideline NSTI	4 (9%)
Other reason, namely:	2 (4%)*

*NSTI* necrotizing soft tissue infection

*Other reasons: ‘answers given were not nuanced enough’, ‘improves wound care’

**Table 5:** Underlying arguments given for the excision of red skin and for non-discolored skin

	Excised red skin (N = 69)	Excised non- discolored skin (N = 12)
This skin contains bacteria that maintain or worsen the infection	20 (29%)	4 (33%)
This skin will eventually turn necrotic, preserving it is pointless	28 (41%)	4 (33%)
This is the way I learned to debride in case of NSTI	25 (36%)	4 (33%)
Other reason, namely:	8* (12%)	2** (17%)

*NSTI* necrotizing soft tissue infection

*Other reason: ‘improves wound care,’ ‘I think it is better to be too cautious than not to be cautious enough’ (2x), ‘I need more information than is given in the case description’ (3x), ‘I no longer agree with my previous given answer,’ ‘other’

**Other reasons: ‘I no longer agree with my previous given answer,’ ‘other’

## Discussion

This is the first study in which practice variation regarding the surgical debridement of NSTI is assessed. An innovative survey was designed in which animated cases were presented. The results show extensive practice variation regarding the amount of skin deemed necessary to be resected upon debridement, ranging from an *en bloc* resection to a skin-sparing approach. Many (half of the respondents) chose an approach in between these extremes. Surgeons working in a hospital with a burn center tended to resect less skin, while plastic surgeons in comparison to general surgeons tended to resect more skin.

Adherence to medical guidelines is a known issue, and it remains under debate whether clinical practice guidelines directly improve clinical practice [16–18]. While clinical practice guidelines may reduce unwanted practice variety by synthesizing and disseminating the best available evidence, they are often based on a homogeneous population, and not always applicable to local settings [17, 18]. Multiple barriers for the adherence to guidelines have been identified, including a lack of awareness, lack of familiarity, lack of agreement, lack of self-efficacy, lack of outcome expectancy, the inertia of previous practice, as well as external barriers [16]. The results of our study indicate noncompliance with the current Dutch guideline for NSTI, in which a skin-sparing approach for the debridement of NSTI is recommended [13, 14]. This advice is based on expert opinion from clinicians well known with the surgical treatment of patients with NSTI, and the view that resecting non-necrotic skin does not improve source control, but does increase morbidity [12, 19, 20]. However, despite early promising results, [21] the superiority (or non-inferiority) of a skin-sparing approach in comparison to a classic *en bloc* approach is not proven, and opponents may fear inadequate source control when using this approach [13]. This latter was indeed mentioned as a reason to not perform a skin-sparing approach in this study, as well as inertia of previous practice (being trained to resect red skin) and lack of outcome expectancy. (Red skin will become necrotic.) In those who did select a skin-sparing approach, most did so because they believed it would lead to smaller skin defect, and only few (one in ten) did so based on the guideline recommendation. So, in order to improve guideline adherence, a combination of scientific evidence increased awareness, and surgical education would be needed.

Among all responders, there was clear agreement on the need to resect necrotic skin. Interestingly, adjacent to resecting necrotic skin, the vast majority also chose to resect blue discolored skin. Blue discolored skin indicates cyanosis of the skin, which may be, but is not necessarily irreversible [22]. According to the principles of skin-sparing debridement, which is to preserve all but evidently necrotic skin, this cyanotic skin may initially be preserved [12]. Most of the responders (70%) who chose to preserve all skin in case one, corresponding with a skin-sparing approach, chose to resect the blue discolored skin in case two. This indicates that most surgeons currently agree on the need to resect blue discolored skin, including the majority of those who use a skin-sparing approach.

The tendency of plastic surgeons, compared to general surgeons, to resect more skin cannot easily be explained. It could be due to the fact that plastic surgeons have more knowledge on reconstructive possibilities compared to general surgeons, which may lead them to resect more easily. Several reasons might explain why surgeons employed in a general hospital with a burn center were more likely to preserve non-necrotic skin. First, since patients are referred to burn centers with extensive skin defects, surgeons employed in burn centers may be more familiar with the short- and long-term challenges of large skin defects. This possibly results in an increased motivation to reduce the wound size early on. Also, responder bias may be present, resulting in surgeons working in burn centers selecting a more skin-sparing approach, since the project this study is part of was initiated in the Dutch burn centers.

Based on gained experience with an animated, interactive study design, we believe there will be adaptation of these types of studies. Many compliments were received from respondents, as well as requests on information on how to develop such a survey. The main learning points that should be considered before initiating such a study, are costs and availability. Designing such a survey is costly (> 10,000 euros in this case), and dedicated involvement of both researchers and clinicians is critical in each step of the process. All mistakes should be identified directly, to prevent the need to make changes later in the process, which will drive the price considerably. Therefore, we would advise to only use such a design if it does have advantages to a text-based survey, and when sufficient funds and time are available.

A major strength of this study is the method used. To our knowledge, no animated, interactive survey has been used before to assess surgical practice variation. Therefore, considerations of the respondents are based on the exact same visuals enhancing the trustworthiness [23].Since a diverse and cross-country population participated, it is likely the results accurately represent the current practice. This study is strengthened by the sufficient sample to test for significant differences, as well as the observation that respondents answered consciously. This latter is indicated by the fact that none of the respondents chose to preserve necrotic skin in case two, as well as the high intra-observer correlation between the answers provided for both cases.

This study contains several limitations as well. Although the interactive animations approach the real-life situation, images still lack important observations (sensory feedback, vitality of tissue upon incision) that may influence decision making. Furthermore, the alternative recruitment strategies used, including contacting contacts of the senior author, and the consequent inability to calculate an exact response rate may limit findings of this study. However, we expect this to not affect the main conclusions, since answers provided by contacts of the senior author resembled those of other participants, and since the estimated response rate is similar to the exact response rate of a part of the sample. Also, the lack of randomization of the order case presentation limits this study, as does the inability to deviate from the proposed answer options. Lastly, the answers provided in both cases were treated as a scale. This scale is, however, not validated, and it is unclear if the distance between the steps is evenly divided.

In conclusion, despite limitations, we believe this study fills a knowledge gap in the current literature on the approach to the debridement of NSTI. It shows extensive variation in current practice, despite guideline recommendations. It thereby emphasizes the need for clinical studies, in which outcomes of different approaches to debridement are registered, in order to achieve evidence for a superior approach of debridement. Since randomized controlled trials are ethically and practically not feasible, an alternative would be a prospective registry. Hereby, it should take into account the possibility that surgeons perform an approach that is neither an *en bloc* nor a skin-sparing approach. In time, this may reveal which approach leads to best outcomes, after which dissemination of results and the facilitation of surgical training may improve adaptation. By repeating this study afterward and comparing with this baseline measurement, the effect of future studies, dissemination and training could even be quantified.

## Supplementary Information

Below is the link to the electronic supplementary material.

Supplementary file1 (PDF 3334 kb)
