# Supplementary material for: Approaches to Surgical Debridement in Necrotizing Soft Tissue Infections: Outcomes of an Animated, Interactive Survey
Source: World J Surg. 2022 Feb 20;46(5):1051–8. doi: 10.1007/s00268-022-06470-8 (PMC8971155; doi:10.1007/s00268-022-06470-8)
Supplement: Supplementary file 1 — Supplementary file1 (PDF 3334 kb) [file 268_2022_6470_MOESM1_ESM.pdf]

Supplementary material for:

Approaches to Surgical Debridement in Necrotizing Soft Tissue Infections: Outcomes of An Animated, Interactive Survey

World Journal of Surgery

Jaco Suijker, MD, MSc, Fabienne A.C. Hofmans, BSc, Paul P.M. van Zuijlen, MD, PhD, Huib A. Cense, MD, PhD, H. Jaap Bonjer, MD, PhD, Annebeth Meij-de Vries, MD, PhD

Corresponding author:

A. Meij-de Vries

Red Cross Hospital Beverwijk, Burn Center

Vondellaan 13, 1942 LE Beverwijk.

T: +31 (0)251 78 49 17 | E: adevries@rkz.nl

Appendix A. Original survey and follow-up questions with translation (Dutch to English)

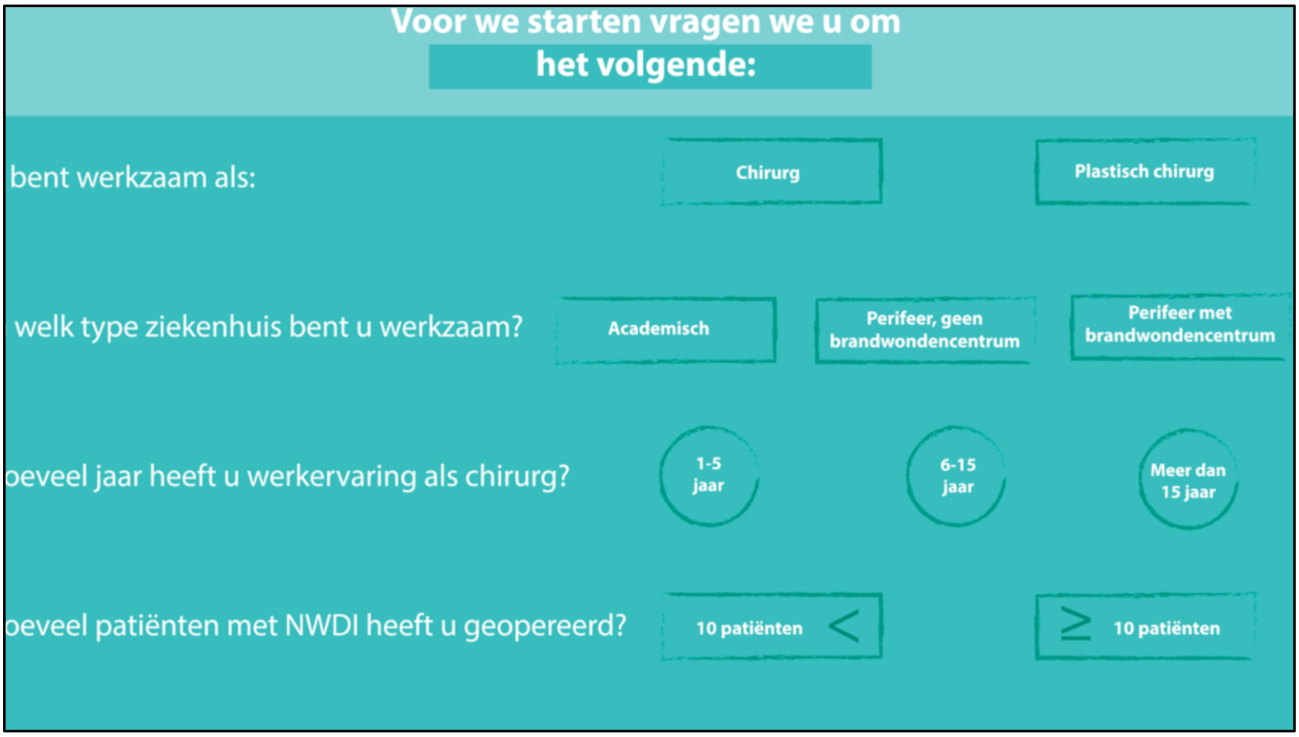

Slide 1
Before we start, we ask you for the following:
You work as a: A: general surgeon B: plastic surgeon
In what type of hospital are you employed? A academic B: peripheral hospital without burns center C: peripheral hospital with burns center
How many years of work experience do you have as a surgeon? A: 1-5 years B: 6-15 years C: >15 years
How many patients with NSTI have you performed surgery on? A: <10 patients B: >10 patients

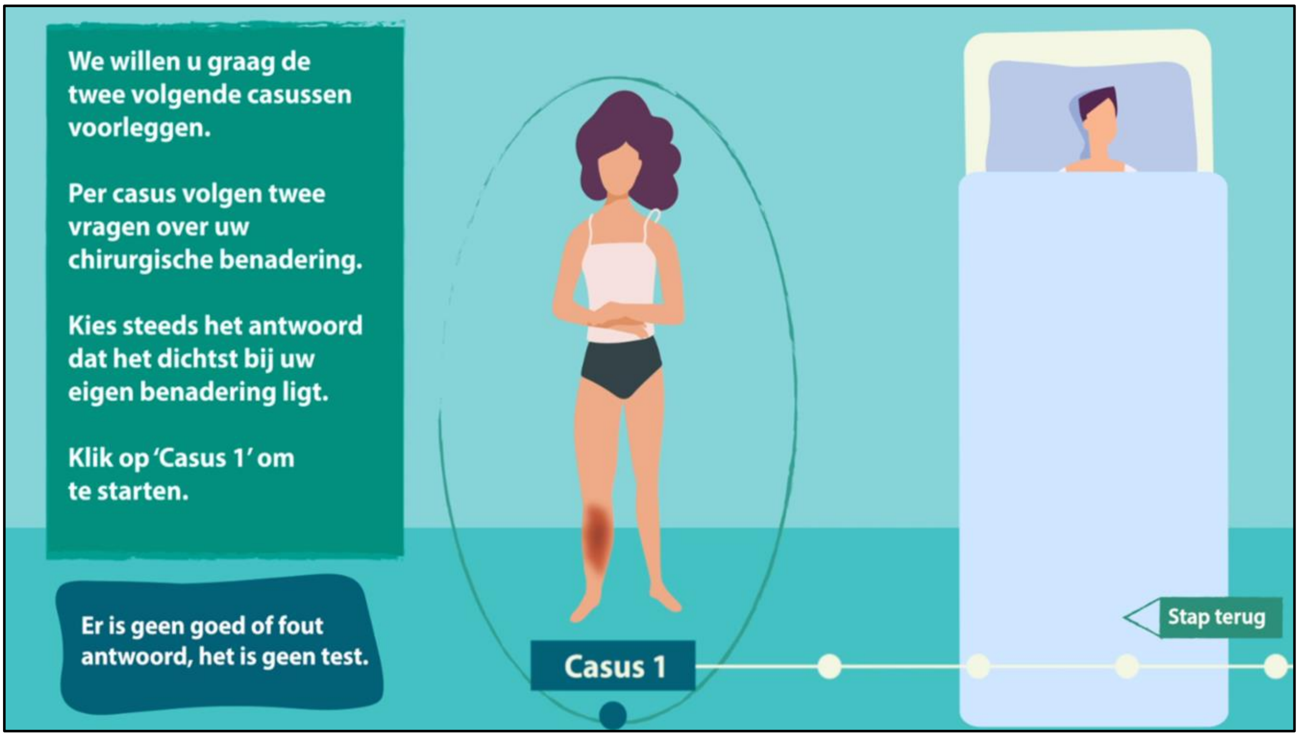

Slide 2
Upper left corner, green text box: We would like to present you the following two cases. In each case two questions regarding chirurgical approach are asked. Always choose the answer that is closest to your own approach. Click on 'casus 1' to start.
Lower left corner, blue tekst box: There is no right or wrong answer, this is not a test.

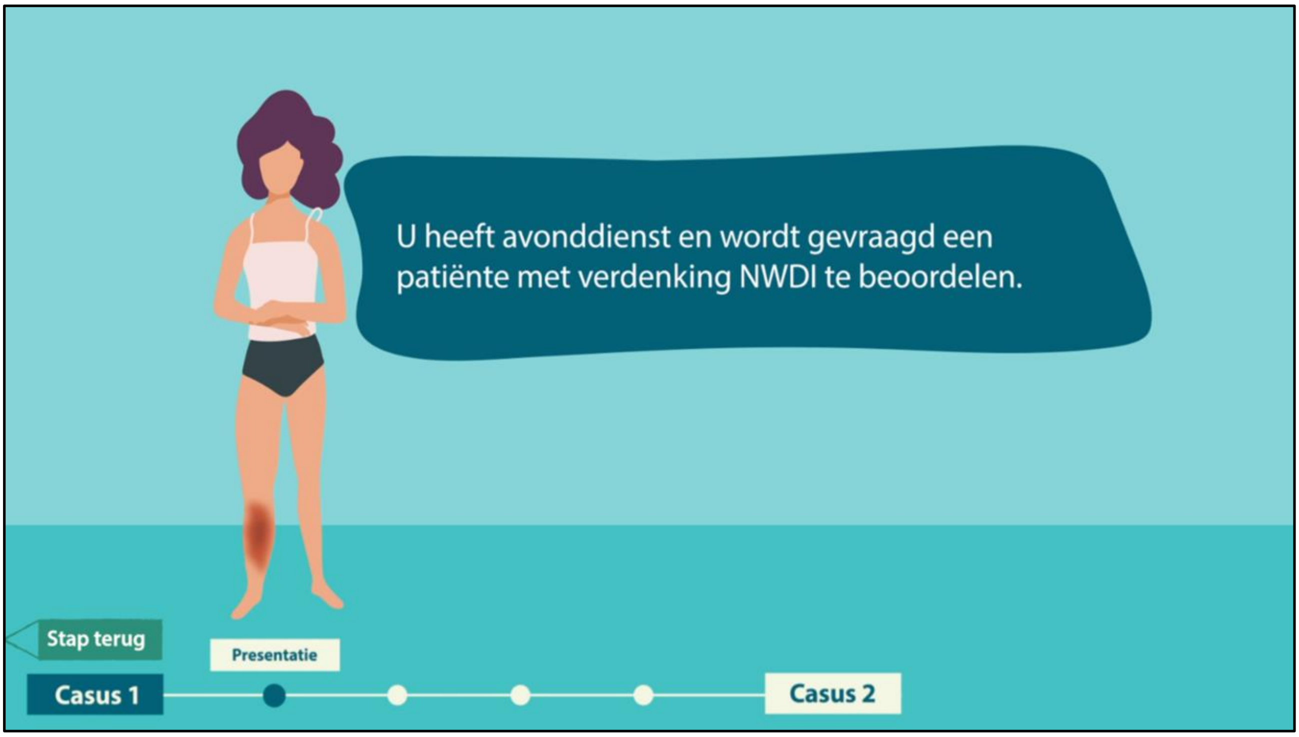

Slide 3
You are on a night shift and are asked to examine a patient suspected to have NSTI.

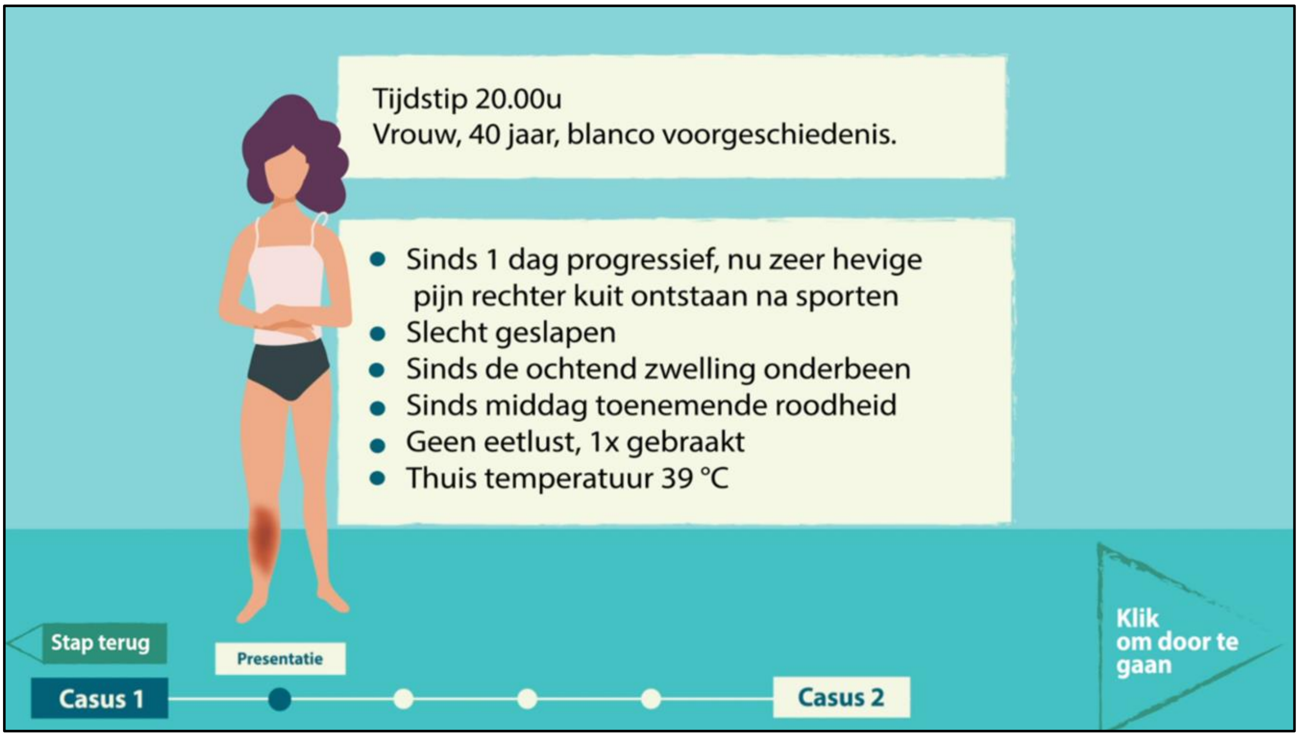

Slide 4
Time: 20.00 (=8 PM)
Female, 40 years, no medical history

- Since 1 day progressive, now inntense pain, of the right calf which arose after excercise
- Slept poorly
- Swollen lower limb since the morning
- Progressive redness since the afternoon
- No appetite, vomited 1x
- Measured temperature at home 39 °C

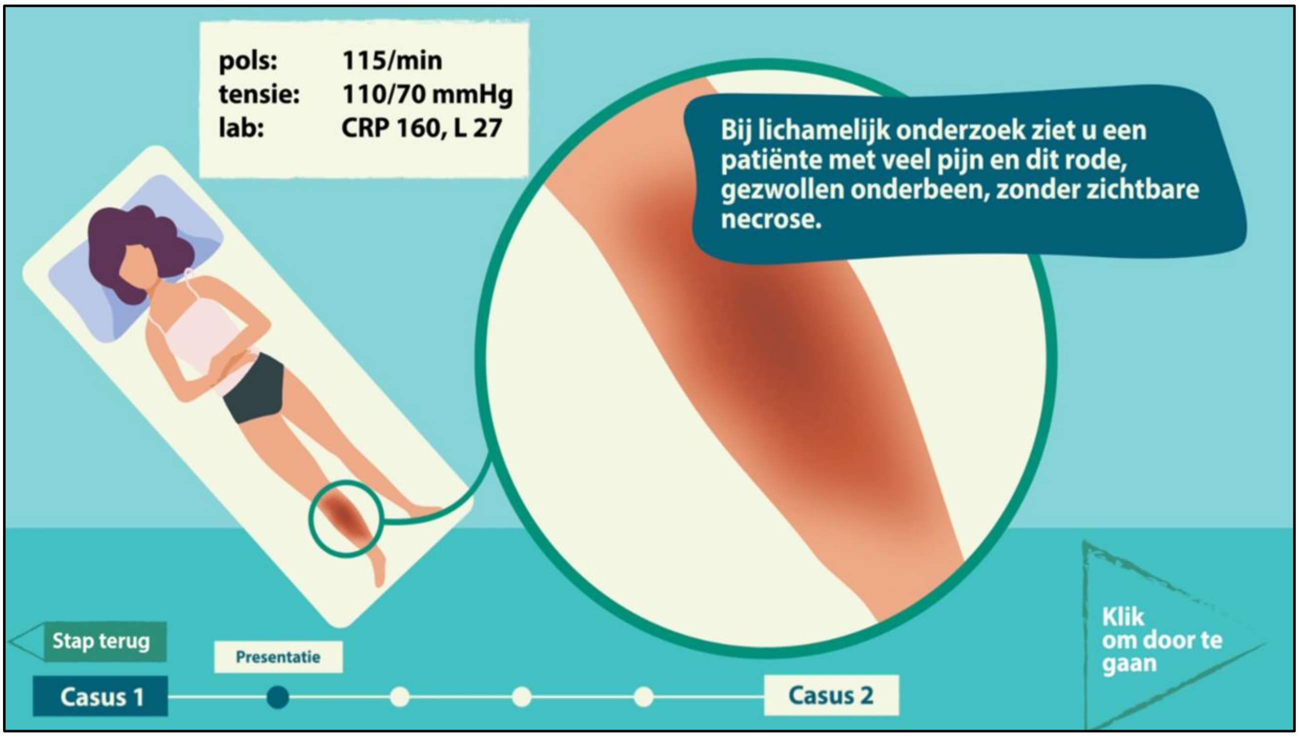

Slide 5
Heart rate: 115/min
Blood pressure: 110/70 mmHg
Lab: CRP 160, L 27

Upon physical examination u see a patient who is in a lot of pain, and has this red, swollen lower limb, without visible necrosis.

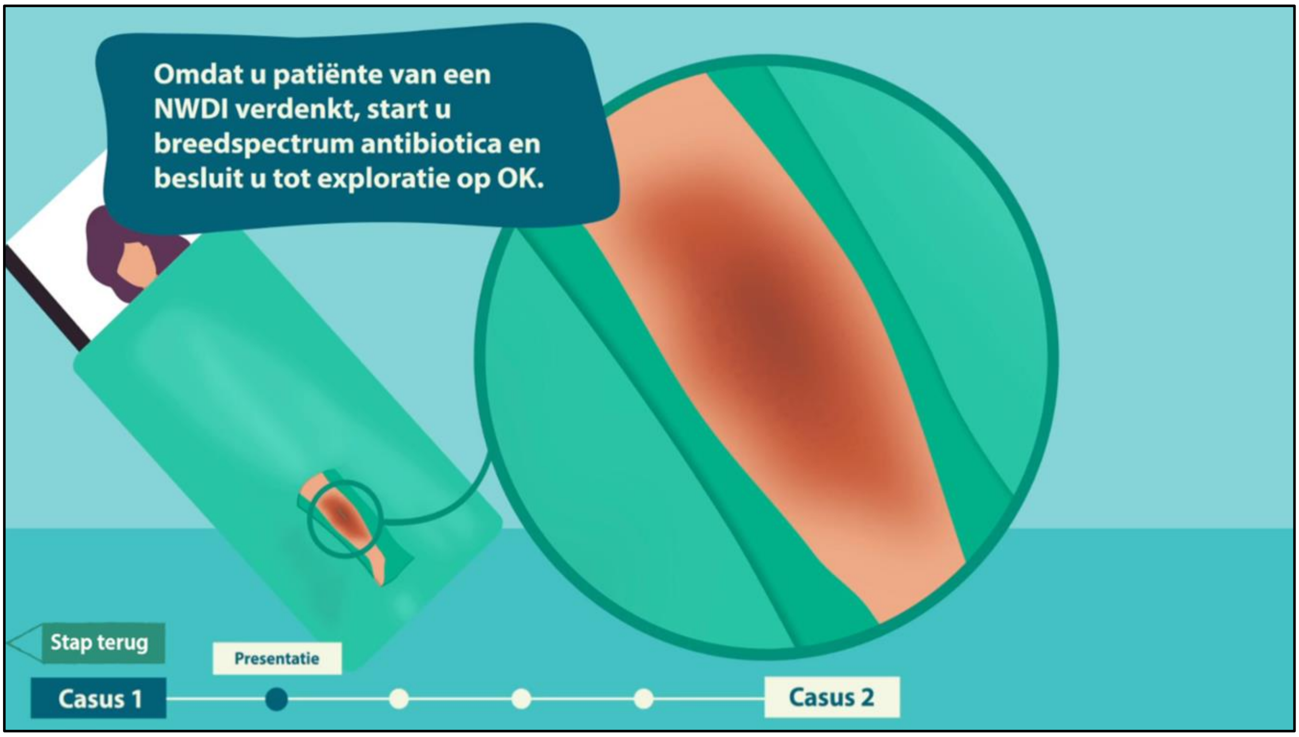

Slide 6
Because you suspect this patient of NSTI, you start broad spectrum antibiotics and decide to perform surgical exploration at the OR.

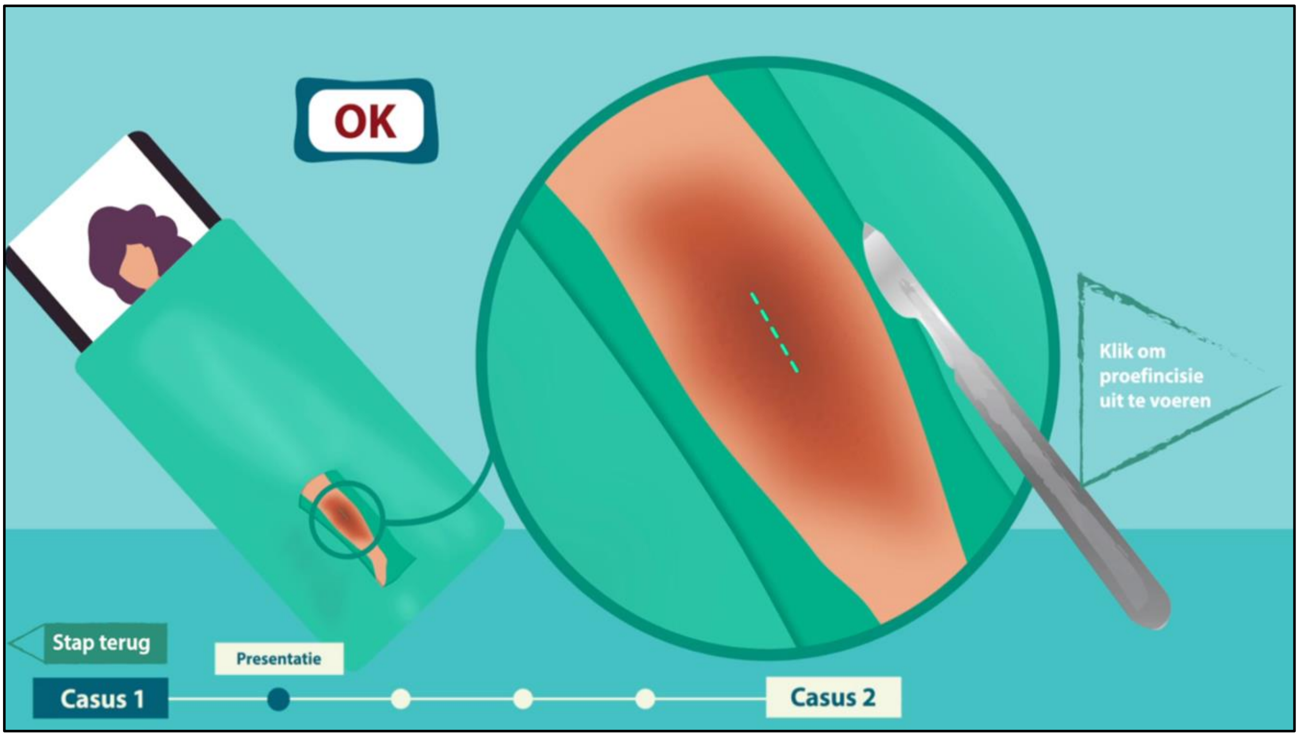

Slide 7
Click to perform a diagnostic incision.

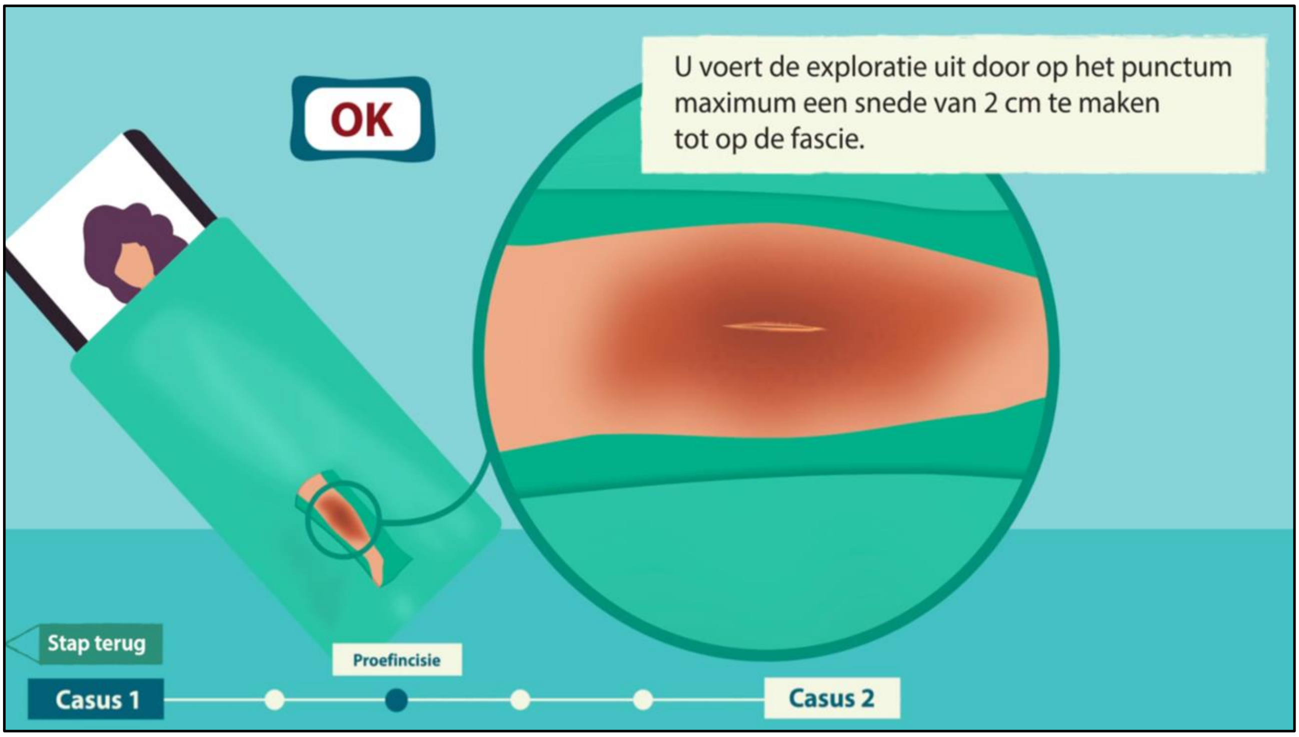

Slide 8
You perform an exploration by making an incision of 2 cm, at the punctum maximum, down to the fascia.

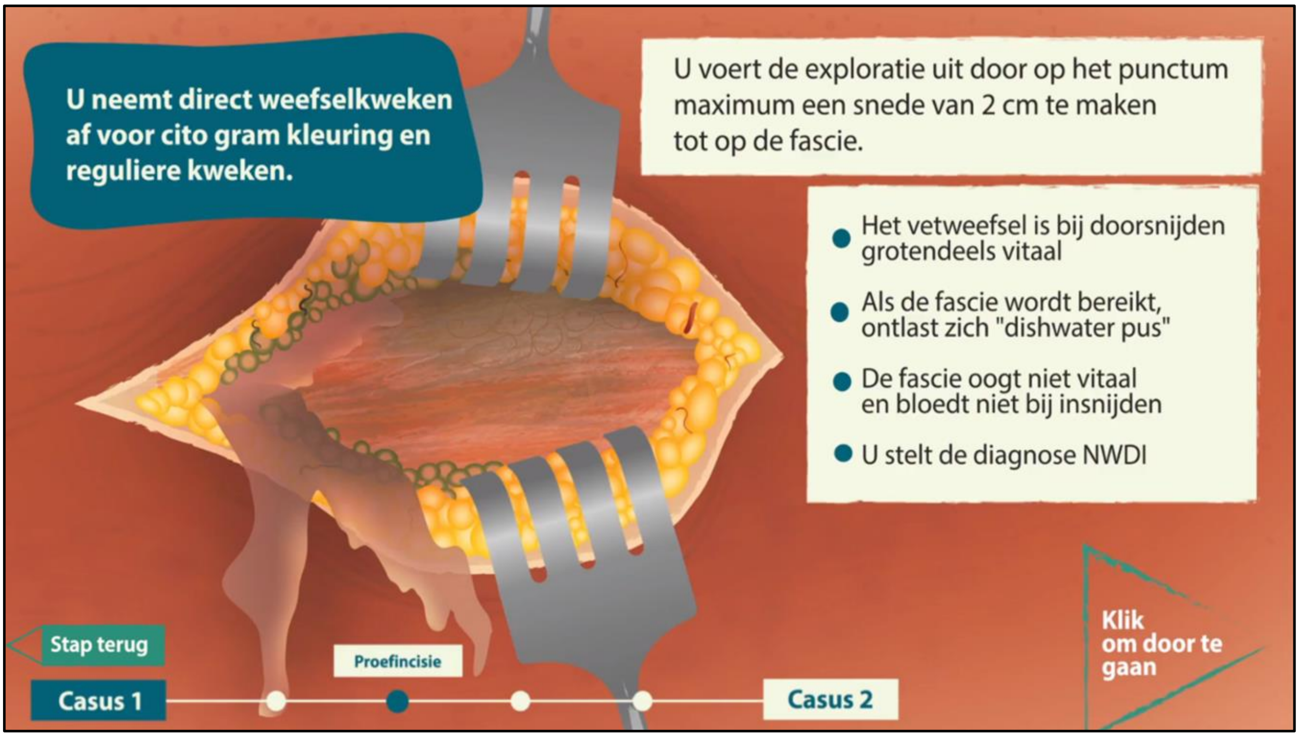

Slide 9

Upper left corner, blue text box: Tissue cultures are taken directly for cito gram staining and regular cultures.

Upper right corner, white text box: You perform an exploration by making an incision of 2 cm, at the punctum maximum, down to the fascia.

Middle right tekst box: * When cutting the fatty tissue this is largely vital
* When reaching the fascia ‘dishwater pus’ relieves itself
* The fascia does not appear vital and does not bleed when cut
* You diagnose the patient with NSTI

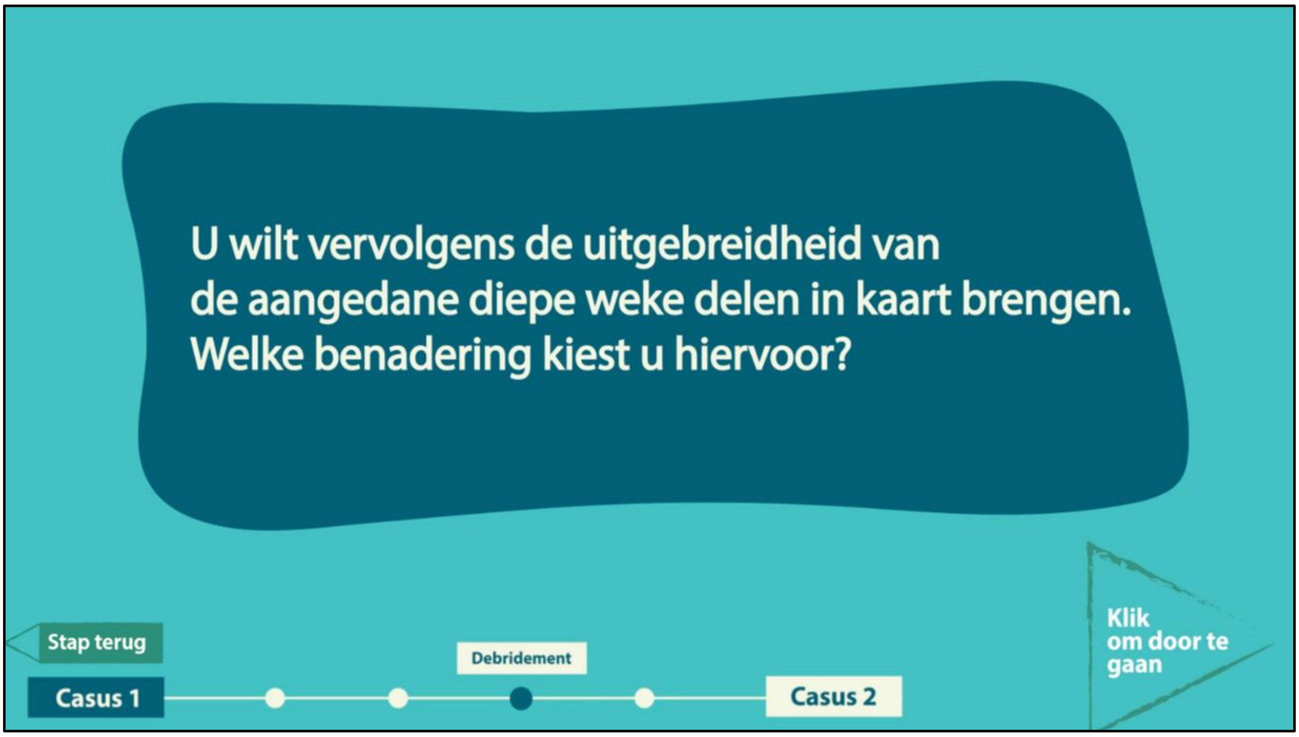

Slide 10
Thereafter you want to explore the extent of the deep soft tissues affected. Which approach do you choose?

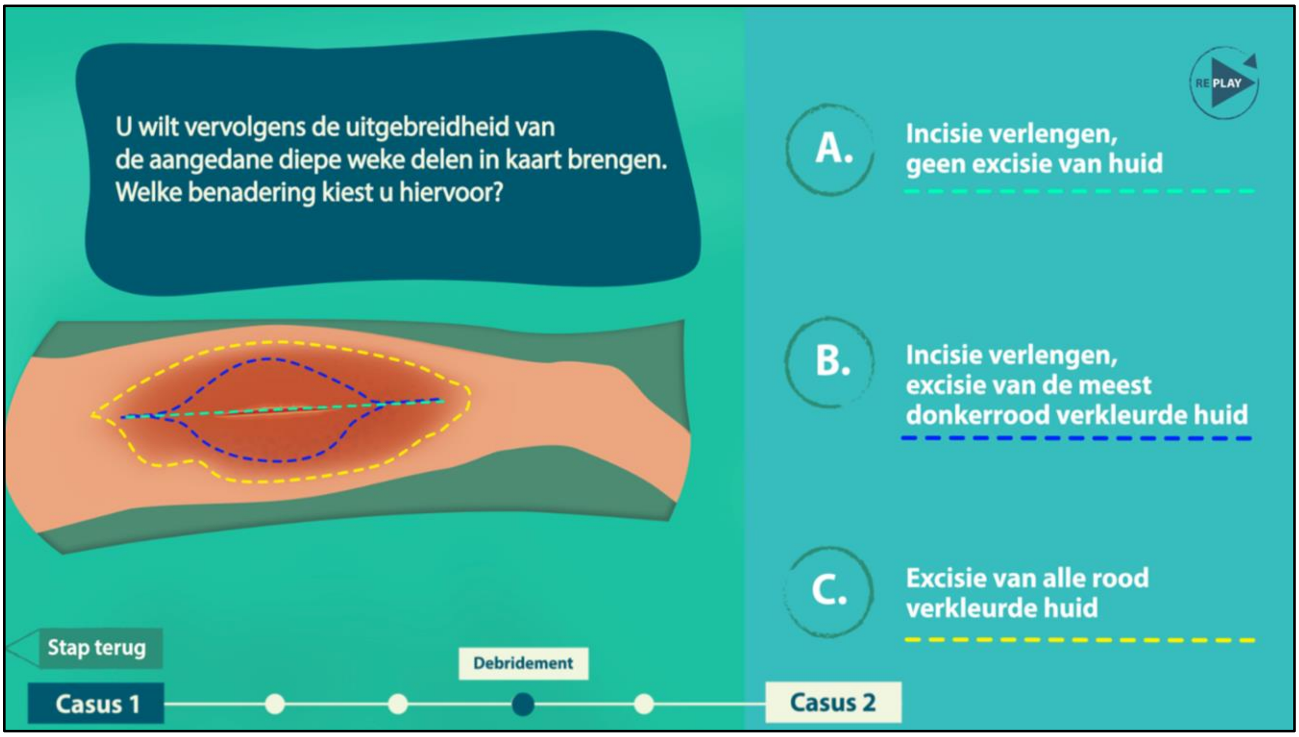

Slide 11
Thereafter you want to explore the extent of the deep soft tissues affected. Which approach do you choose?

A: Incision elongation, no excision of the skin
B: incision elongation, excision of the darkest red discolored skin
C: Excision of all red discolored skin

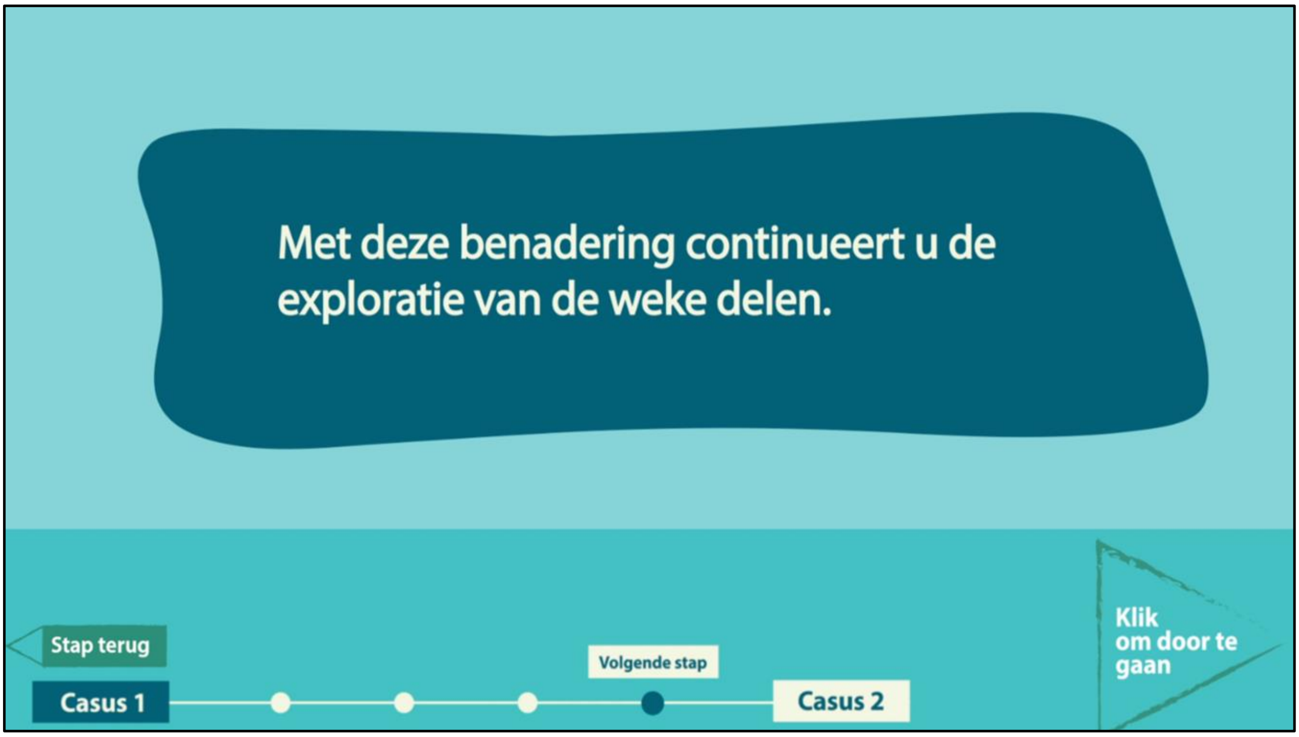

Slide 12
With this approach you continue the exploration of the soft tissues.

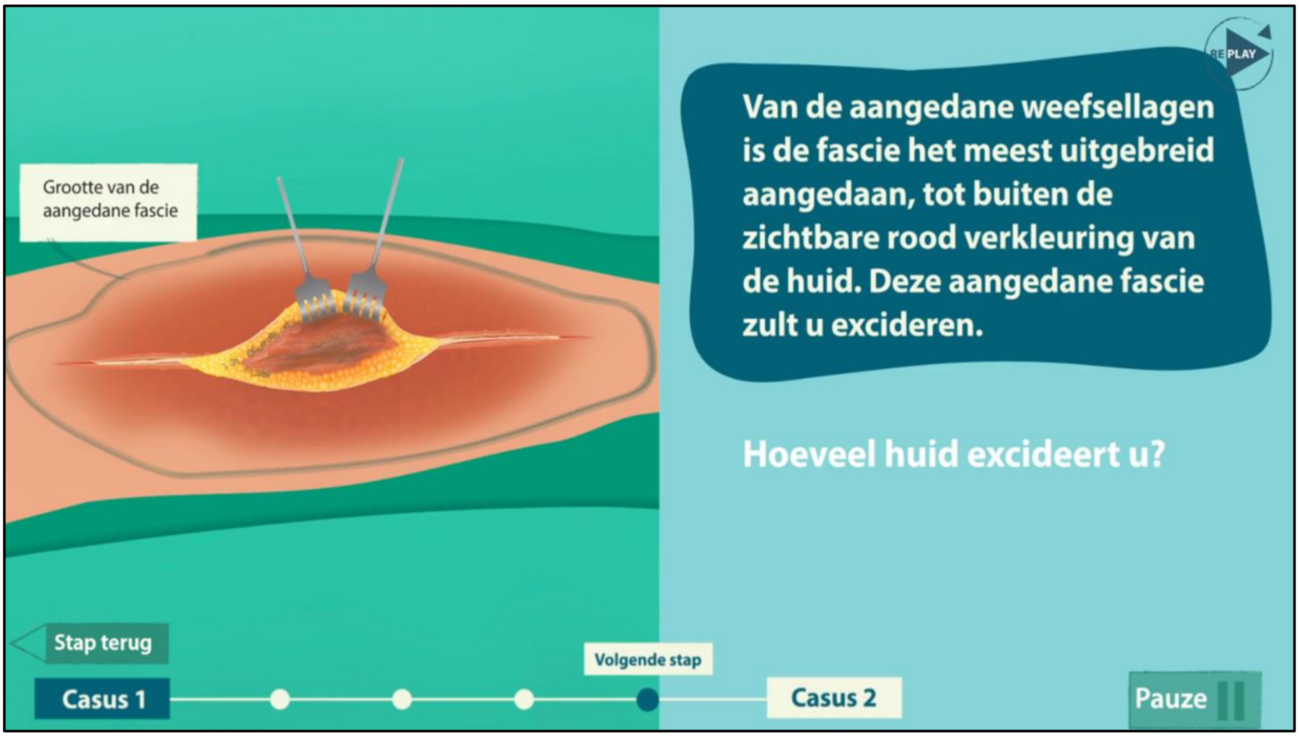

Slide 13
Left middle, white text box: Extend of the affected fascia
Right upper corner, blue text box: Of the affected tissue layers the fascia is the most extensively affected, beyond the visible red discoloration of the skin. You will excise this affected fascia.
Question below the blue text box: How much skin do you excise?

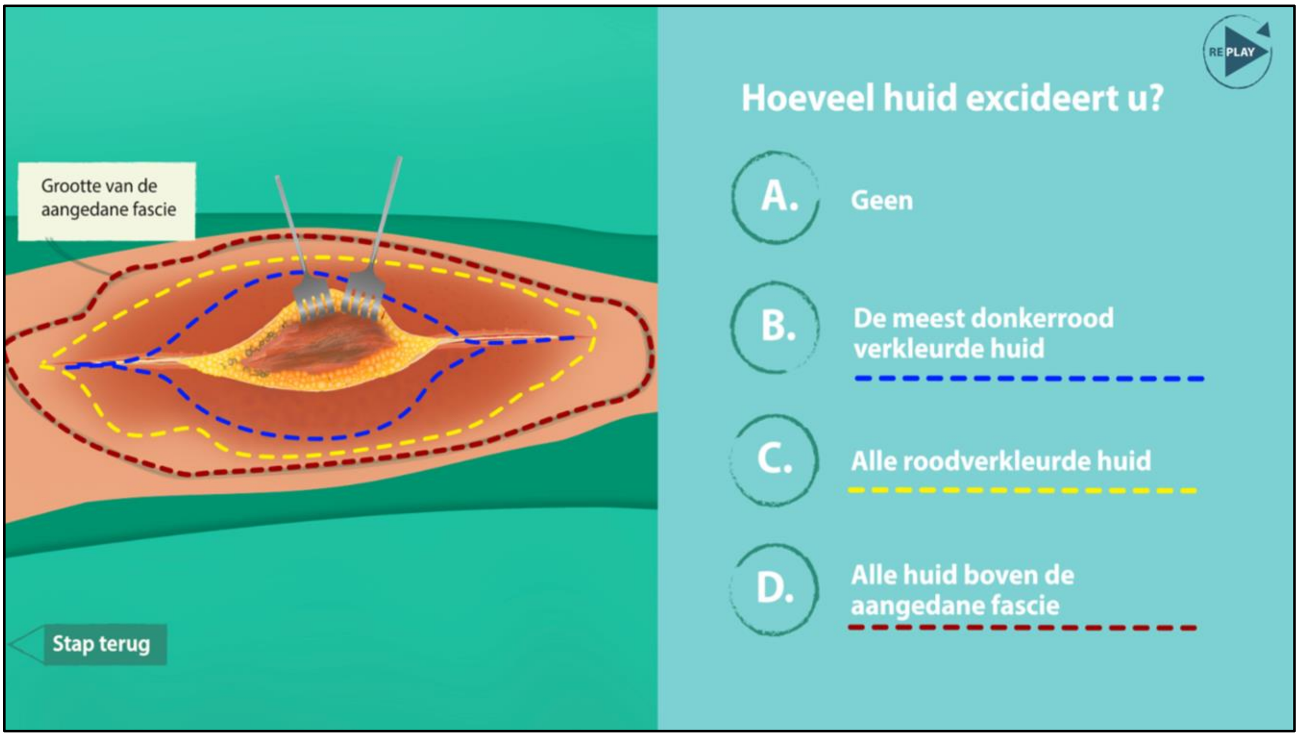

Slide 14A (if answered A to question on slide 11)
Left middle white text box: Extend of the affected fascia
Question right upper corner: How much skin do you excise?

A: None
B: The darkest red discolored skin
C: All red discolored skin
D: All skin above the affected fascia

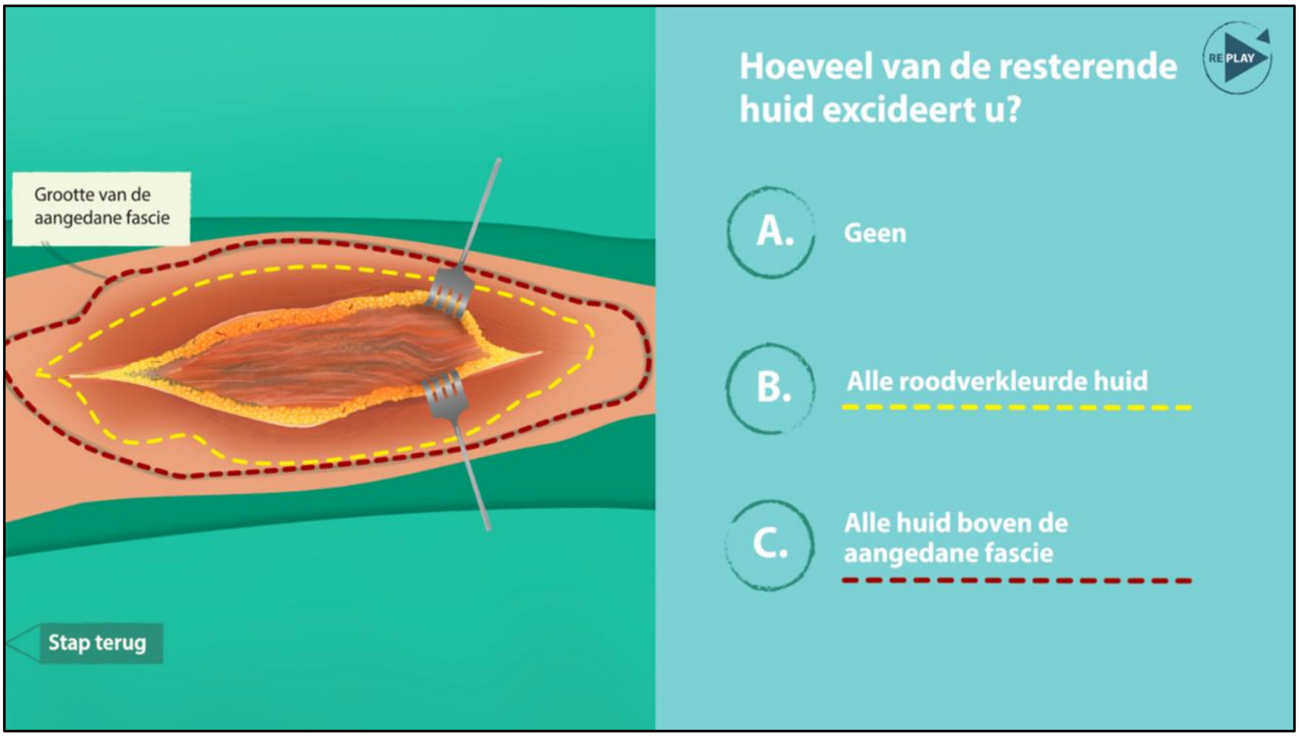

Slide 14B (if answered B to question on slide 11)
Left middle white text box: Extend of the affected fascia
Question right upper corner: How much skin do you excise?

A: None
B: All red discolored skin
C: All skin above the affected fascia

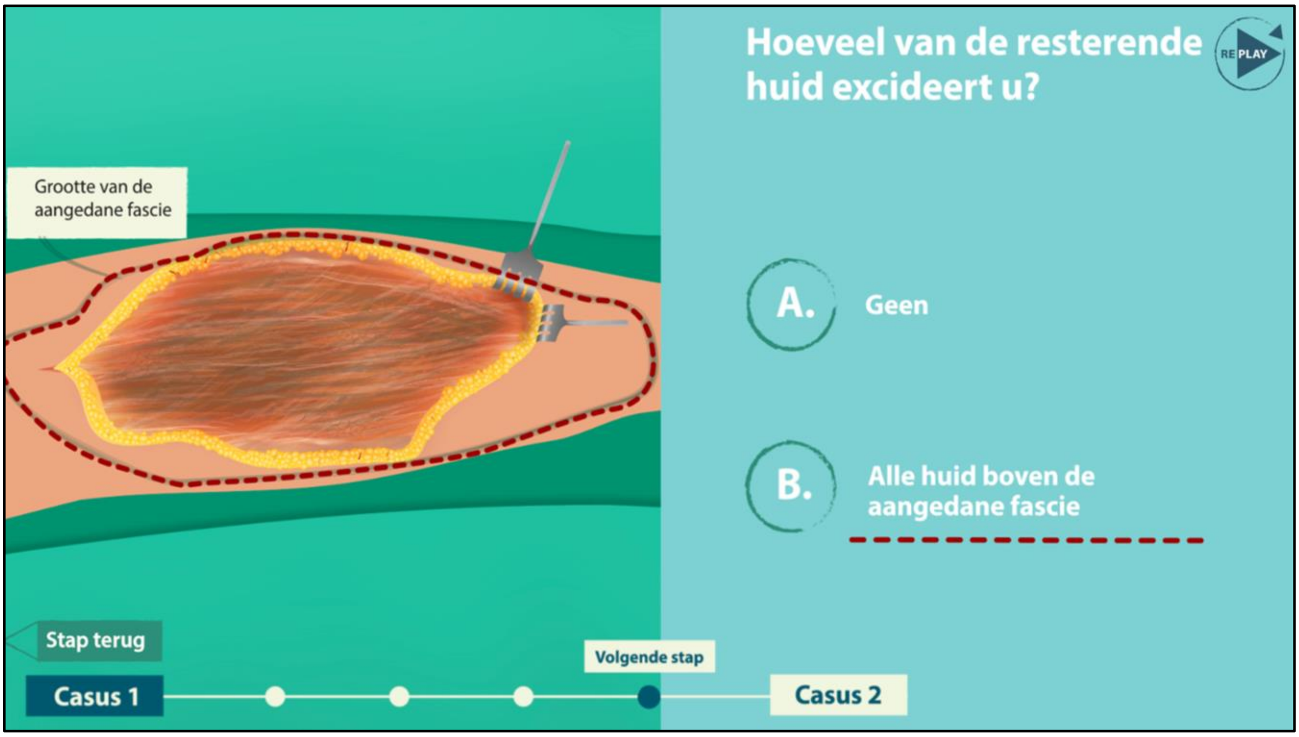

Slide 14C (if answered C to question on slide 11)
Left middle white text box: Extend of the affected fascia
Question right upper corner: How much skin do you excise?

A: None
B: All skin above the affected fascia

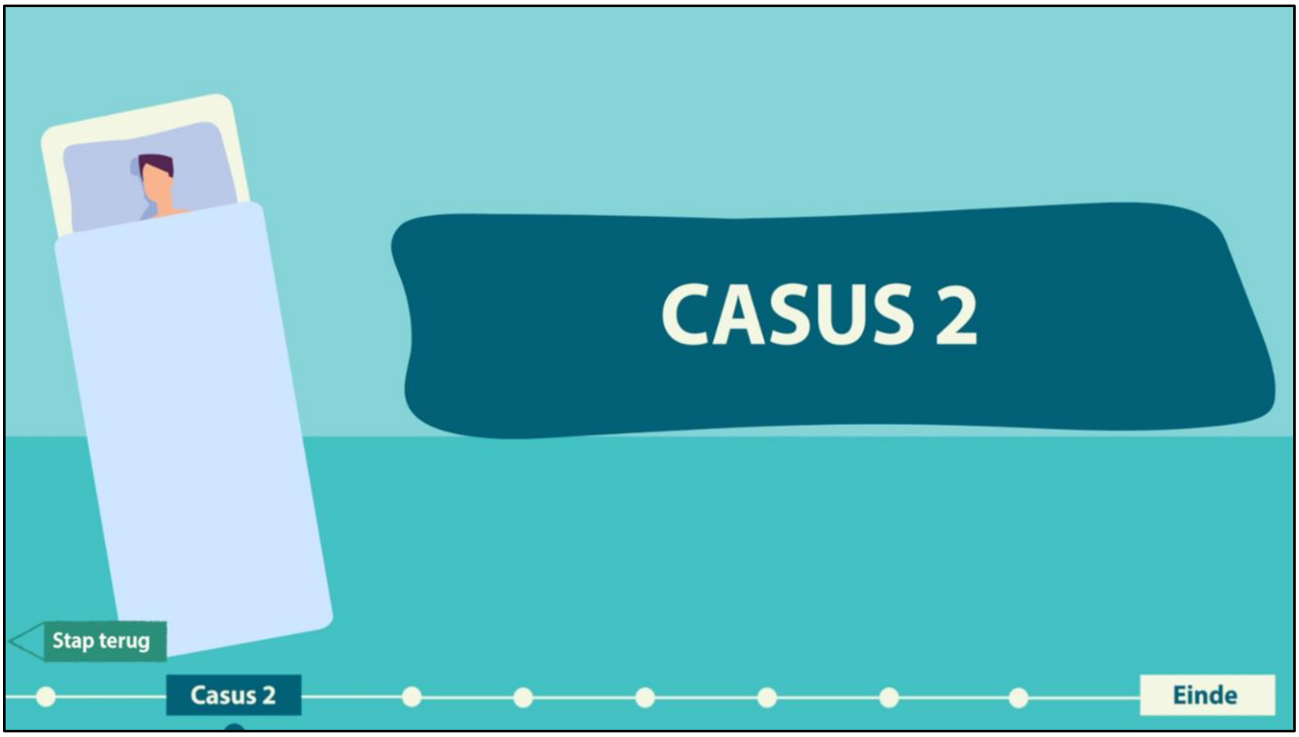

Slide 15

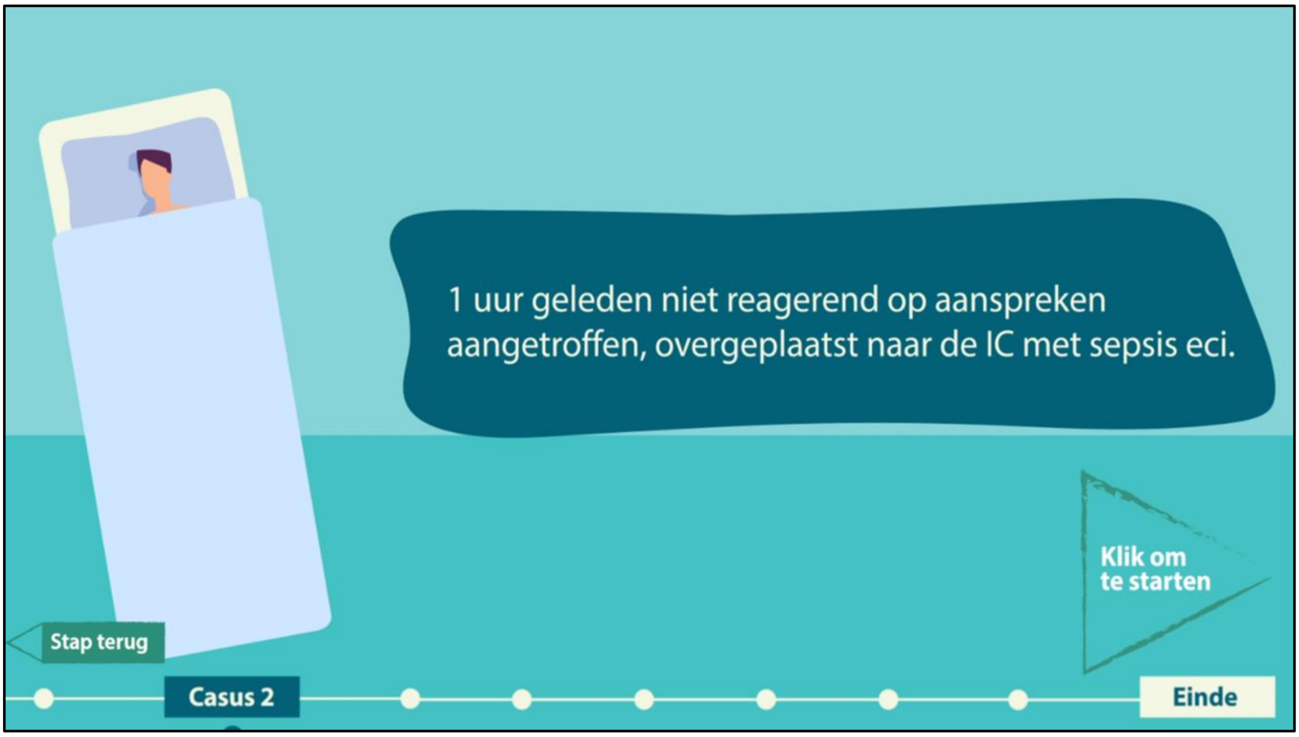

Slide 16
Since 1 hour unresponsive when talked to, transferred to the ICU with sepsis eci

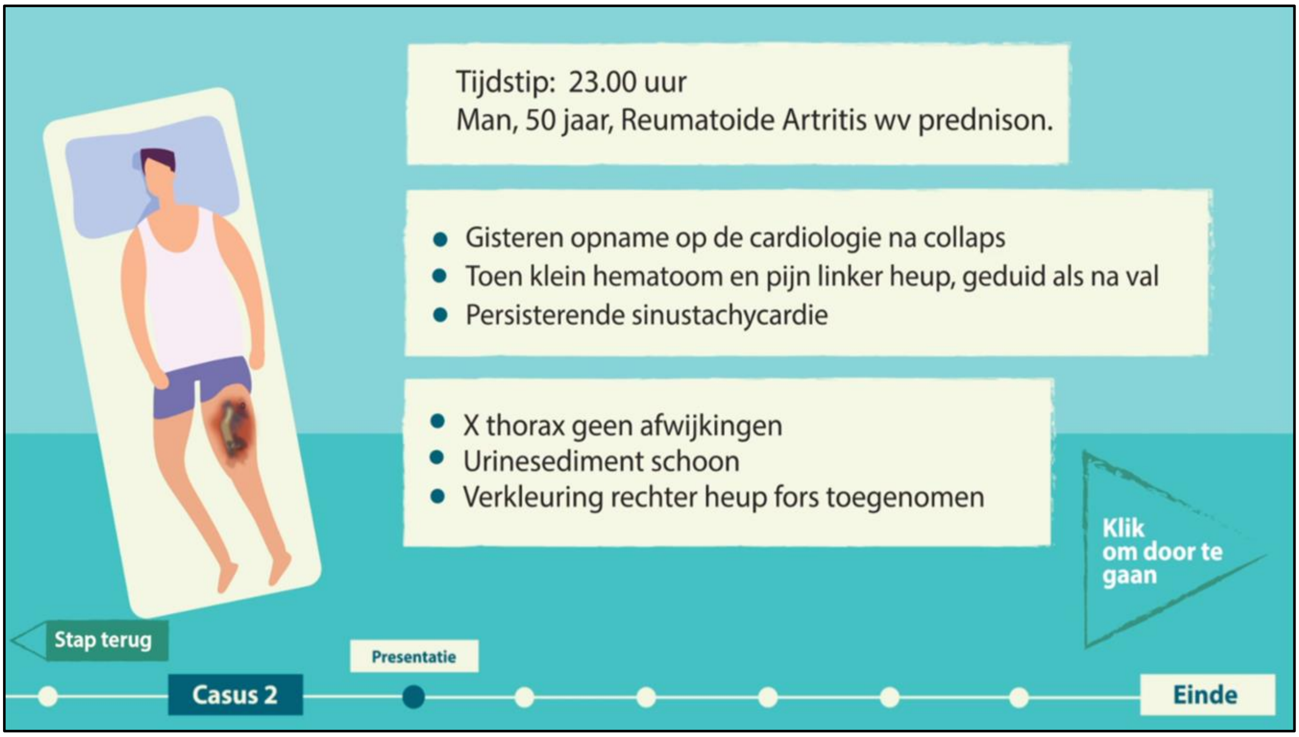

Slide 17
Time: 23.00 (11 PM)
Male, 50 years, rheumatoid arthritis for which prednison

- Yesterday admitted to the cardiology department after collaps
- Then a small hematoma and pain in left hip, interpreted as after fall
- Persisting sinustachycardia

- X-ray of the thorax without abnormalities
- Urine sediment clean
- Discoloration on the right hip increased significantly

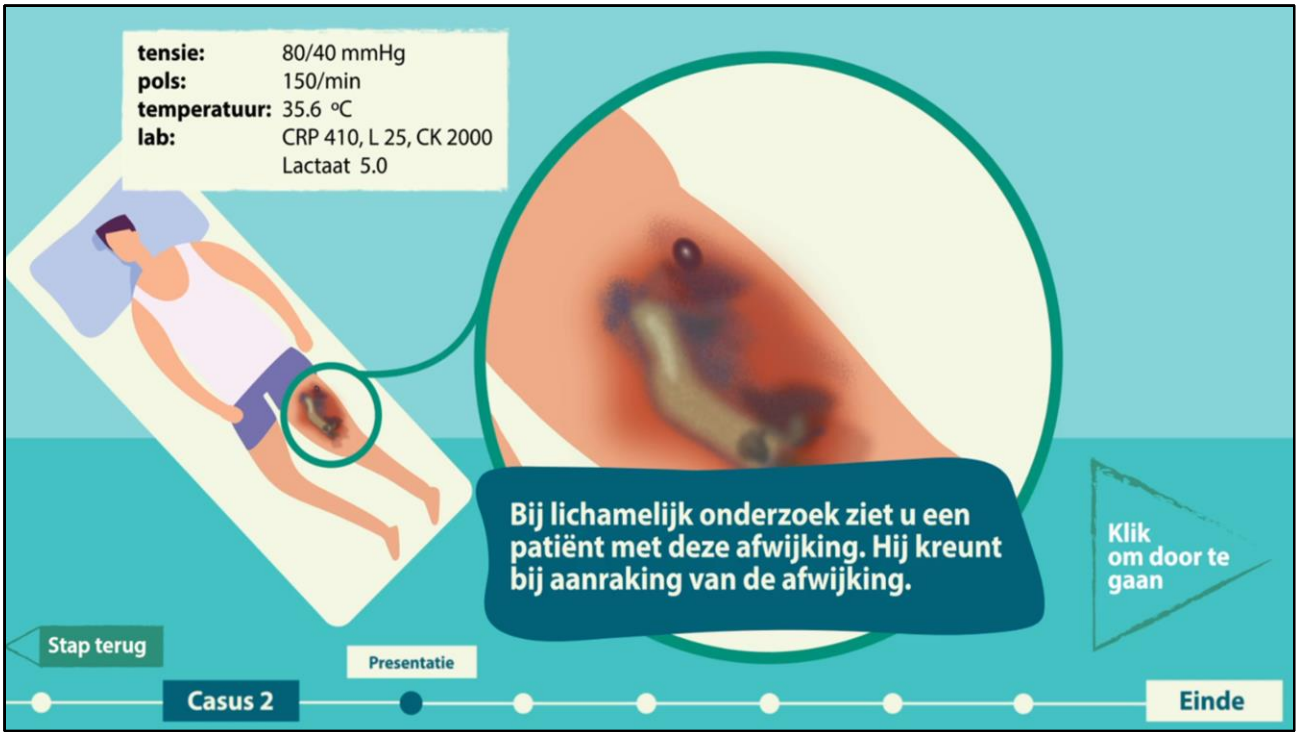

Slide 18
Blood pressure: 80/40 mmHg
Heart rate: 150/min
Temperature: 35.6 °C
Lab: CRP 410, L 25, CK 2000 Lactic acid 5.0

Blue tekst box: On physical examination you see a patient with this abnormality. The patient moans when touching the abnormality.

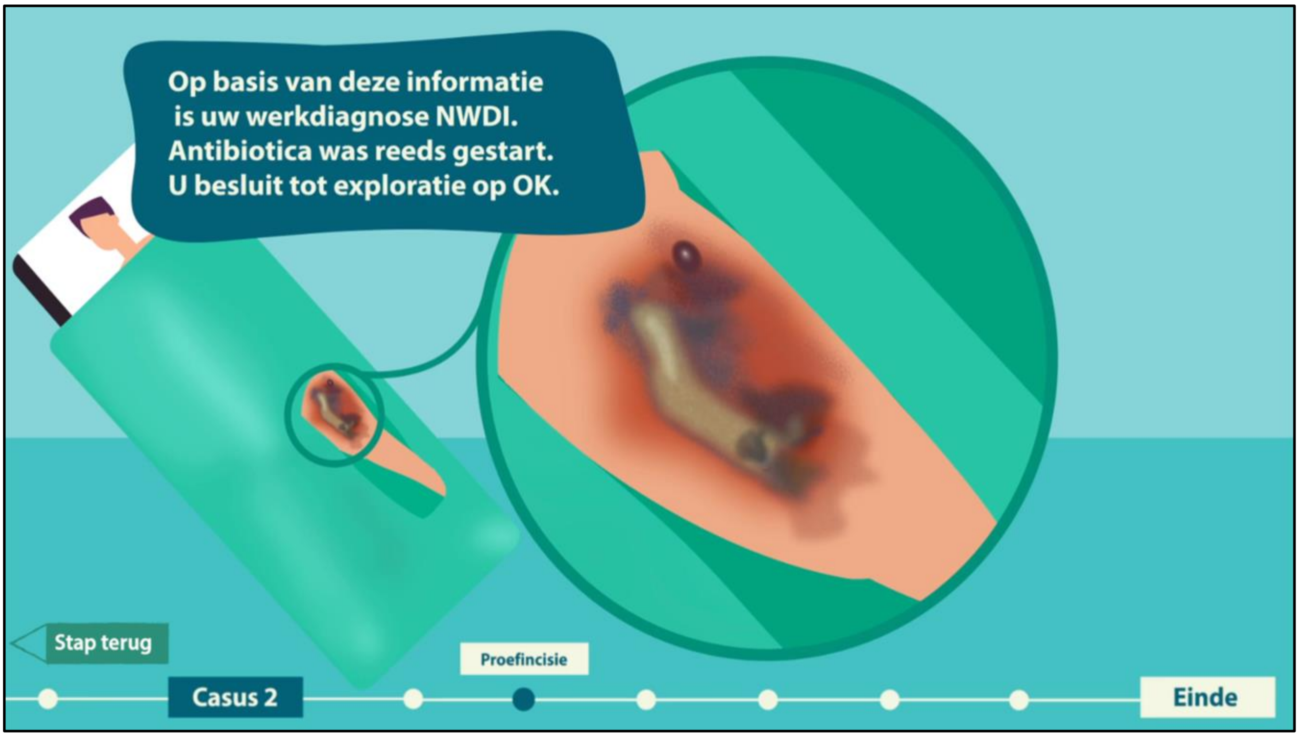

Slide 19
Based on this information your working diagnosis is NSTI. Antibiotics had already been started. You decide to perform surgical exploration at the OR.

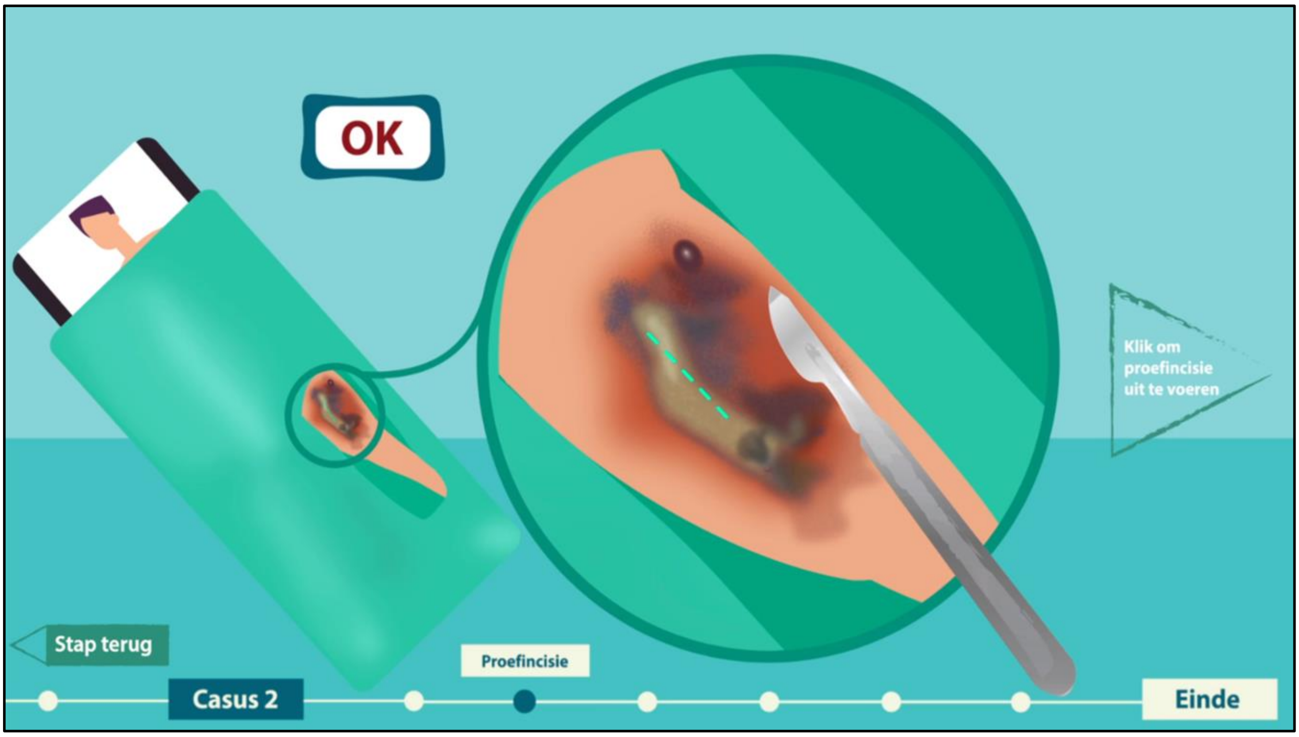

Slide 20
Click to perform a diagnostic incision.

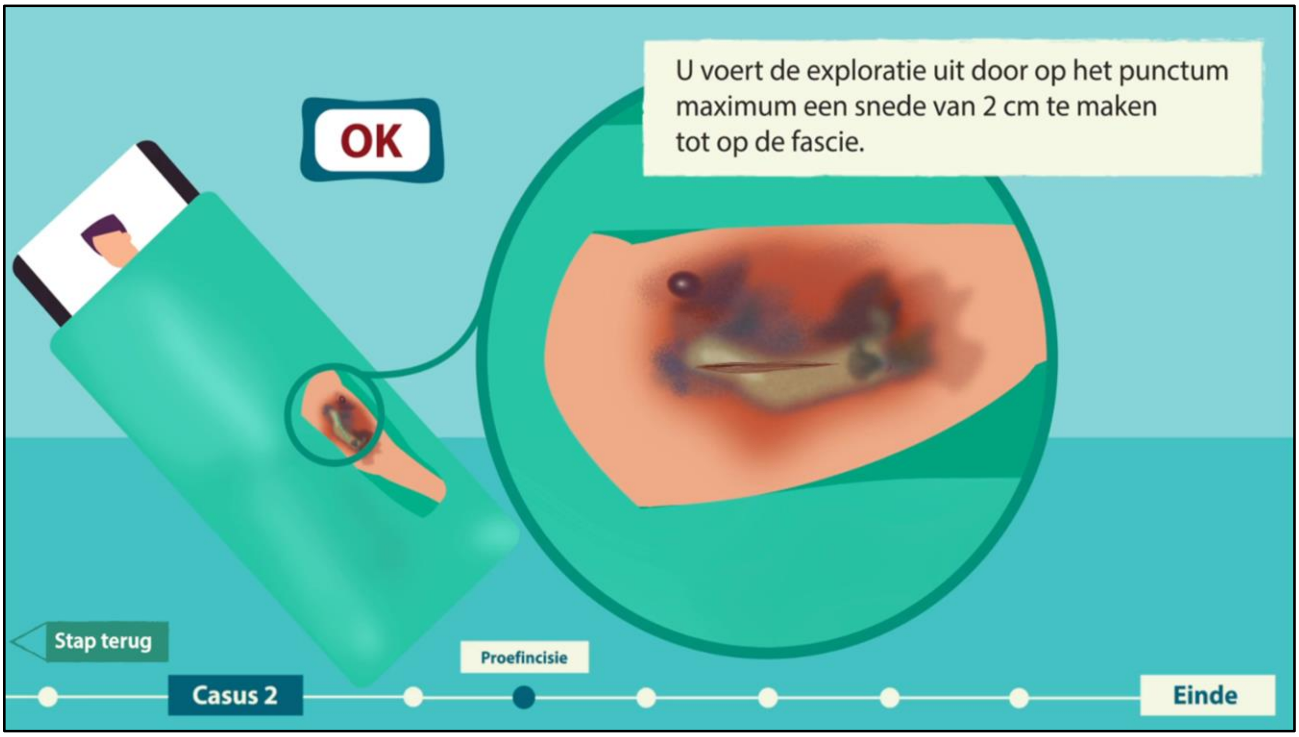

Slide 21
You perform an exploration by making an incision of 2 cm, at the punctum maximum, down to the fascia.

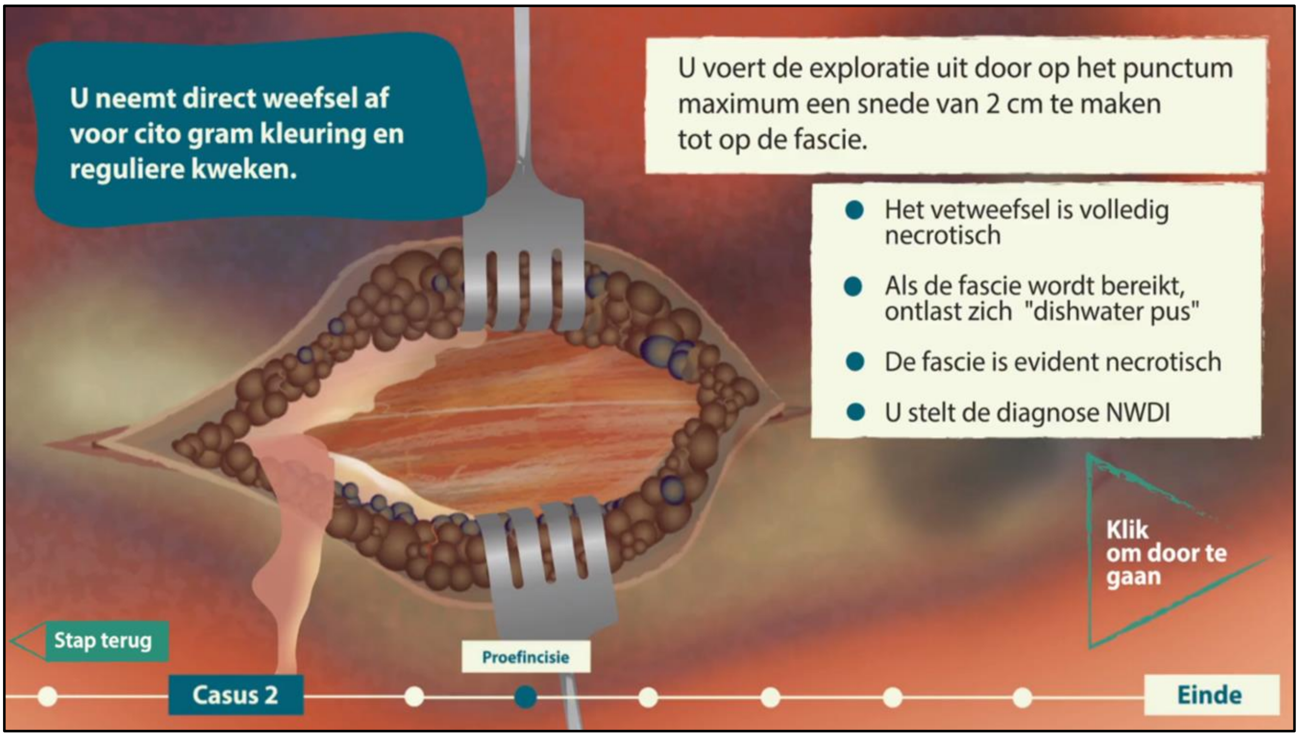

Slide 22
Upper left corner, blue text box: Tissue cultures are taken directly for cito gram staining and regular cultures.
Upper right corner, white text box: You perform an exploration by making an incision of 2 cm, at the punctum maximum, down to the fascia.
Middle right tekst box: * When cutting the fatty tissue it is completely necrotic
* Reaching the fascia ‘dishwater pus’ relieves itself
* The fascia is evidently necrotic
* You diagnose the patient with NSTI

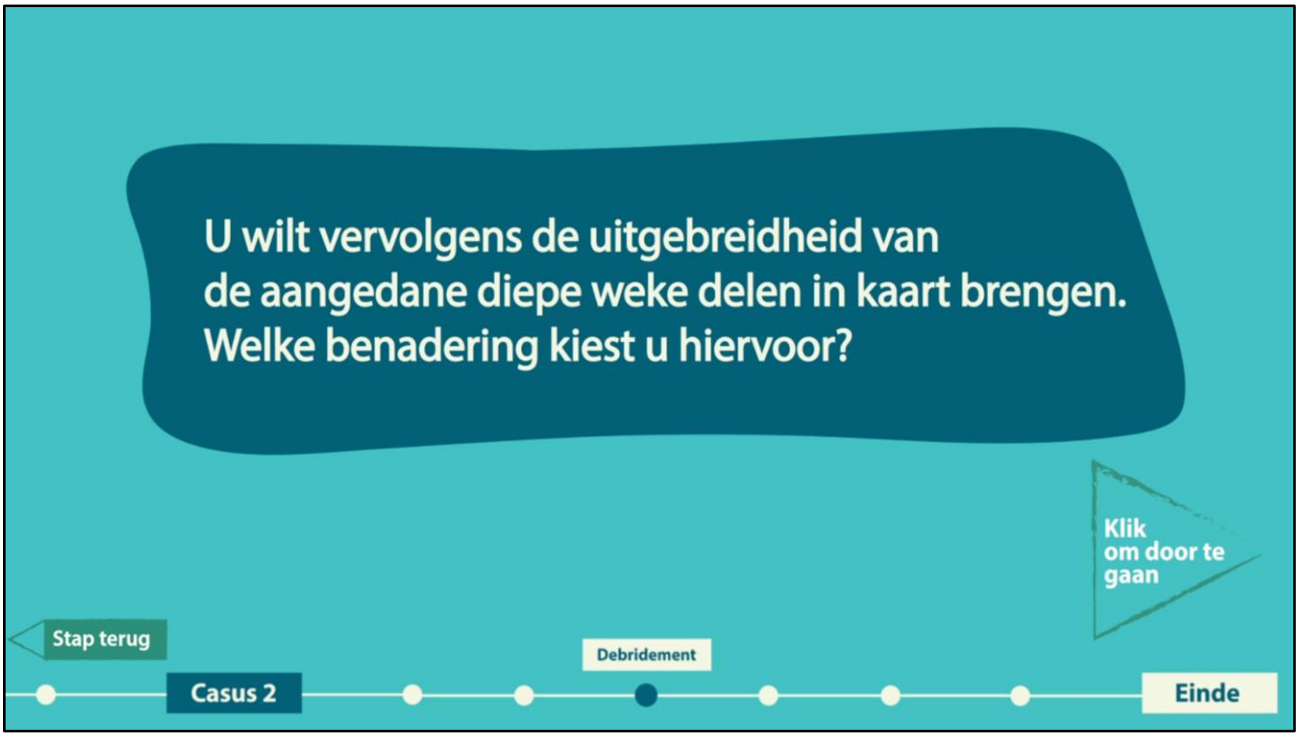

Slide 23
Thereafter you want to explore the extent of the deep soft tissues affected. Which approach do you choose?

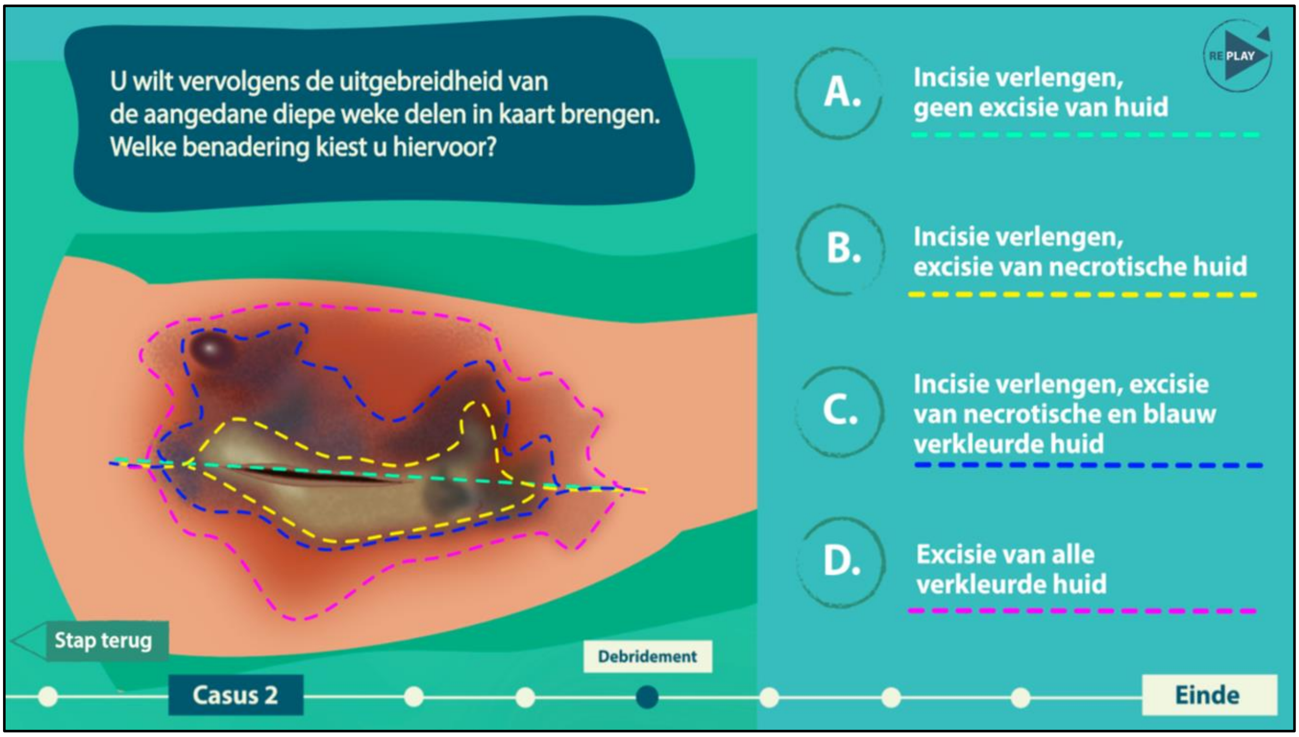

Slide 24

Upper left blue tekst box: Thereafter you want to explore the extent of the deep soft tissues affected. Which approach do you choose?

A: Incision elongation, no excision of the skin
B: incision elongation, excision of the necrotic skin
C: Incision elongation, excision of necrotic and blue discolored skin
D: Excision of all discolored skin

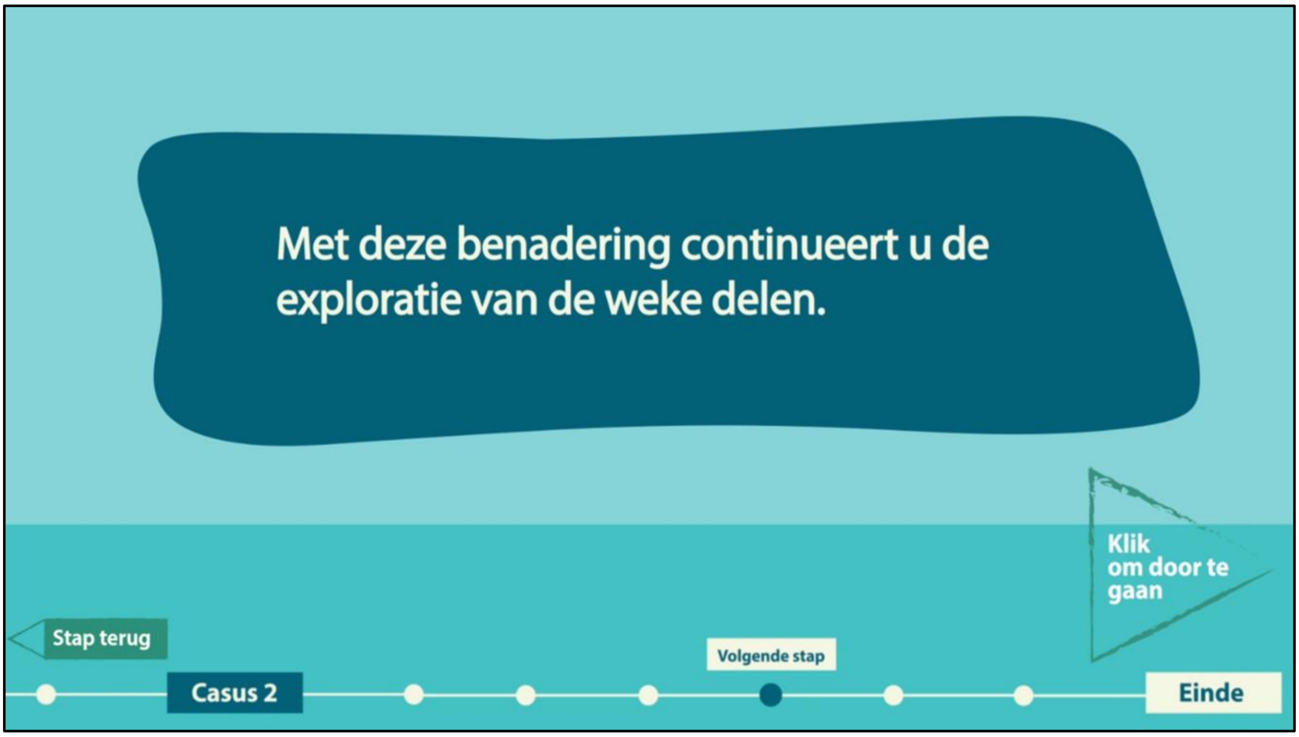

Slide 25
With this approach you continue the exploration of the soft tissues.

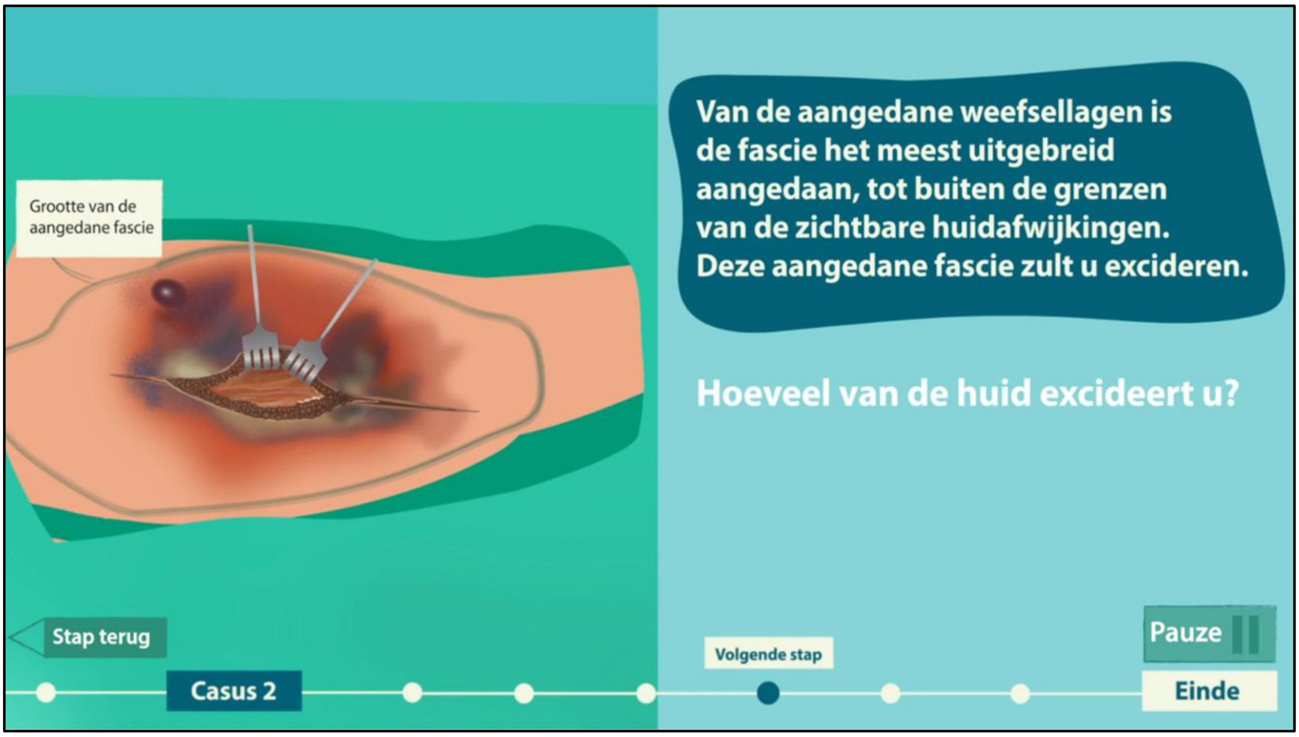

Slide 26
Left middle white text box: Extend of the affected fascia
Right upper corner, blue tekst box: Of the affected tissue layers the fascia is the most extensively affected, beyond the visible red discoloration of the skin. You will excise this affected fascia.
Question below the blue tekst box: How much of the skin do you excise?

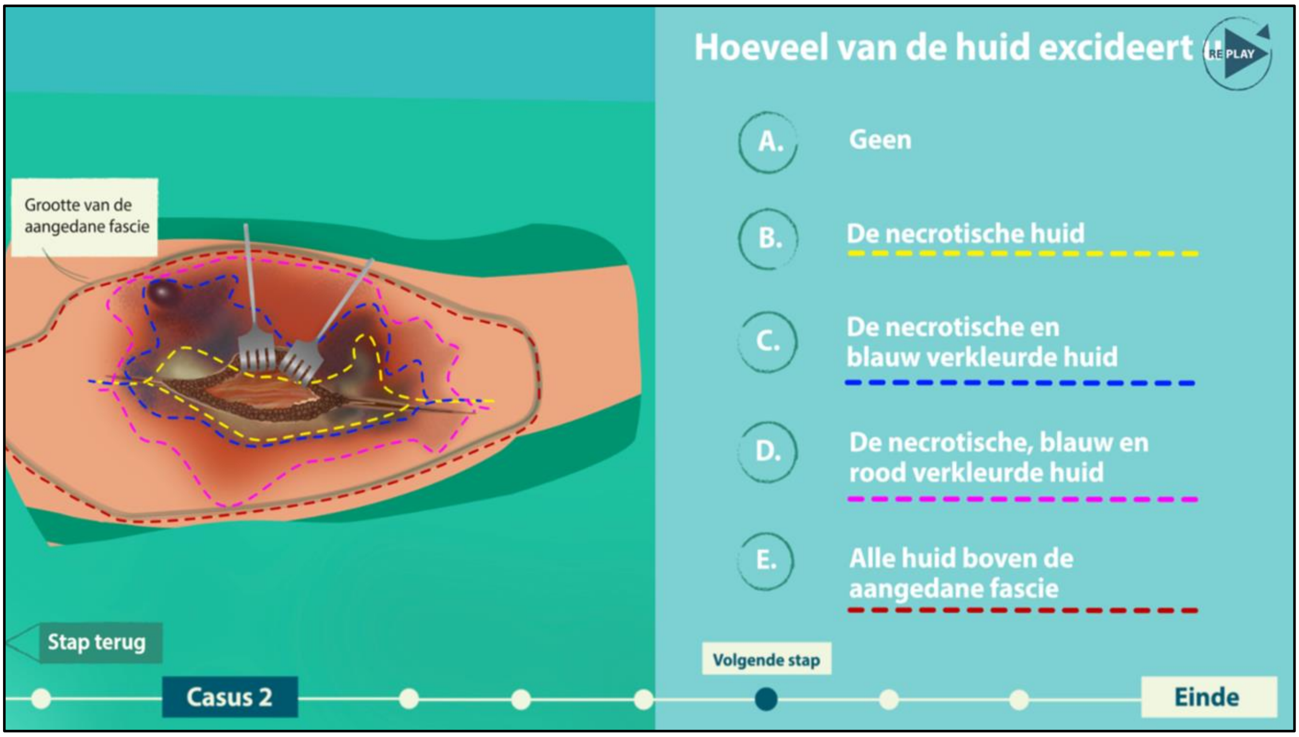

Slide 27A (if answered A to question on slide 24)
Question right upper corner: How much skin do you excise?

A: None
B: The necrotic skin
C: The necrotic and blue discolored skin
D: The necrotic, blue and red discolored skin
E: All skin above the affected fascia

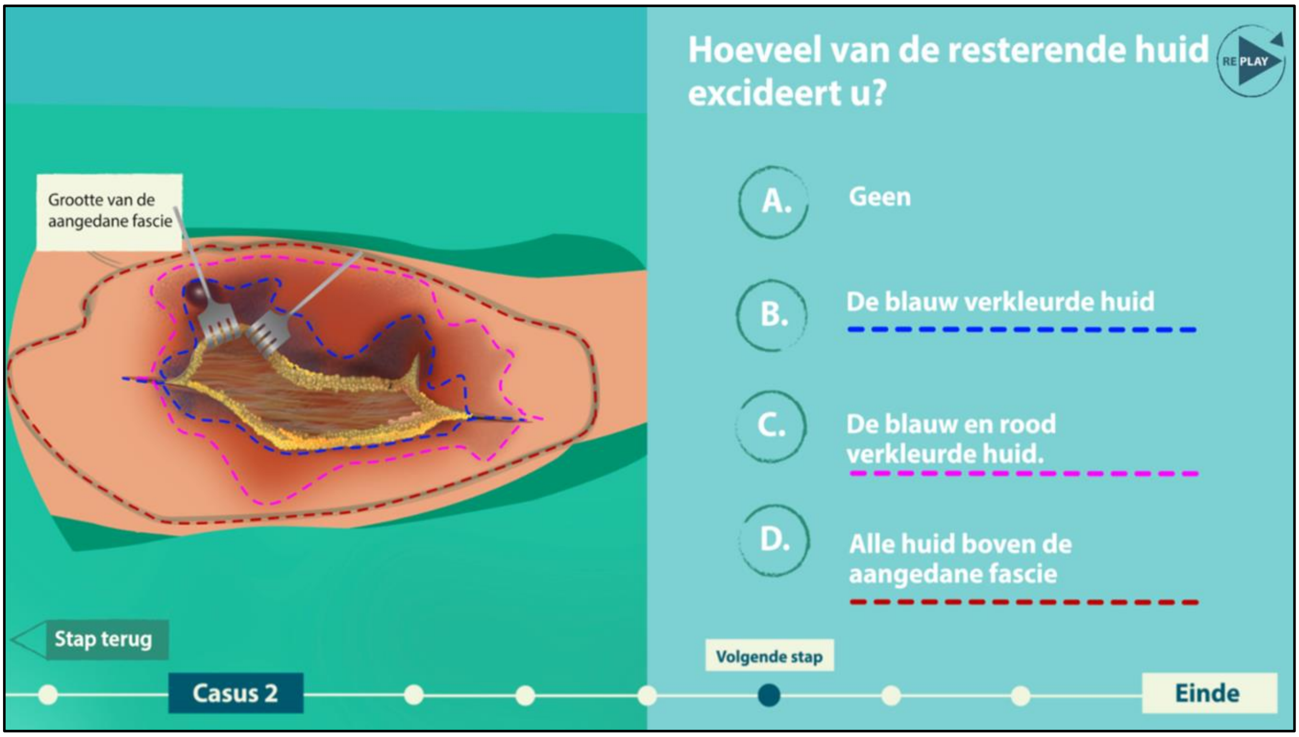

Slide 27B (if answered B to question on slide 24)
Question right upper corner: How much skin do you excise?

A: None
B: The blue discolored skin
C: The blue and red discolored skin
D: All skin above the affected fascia

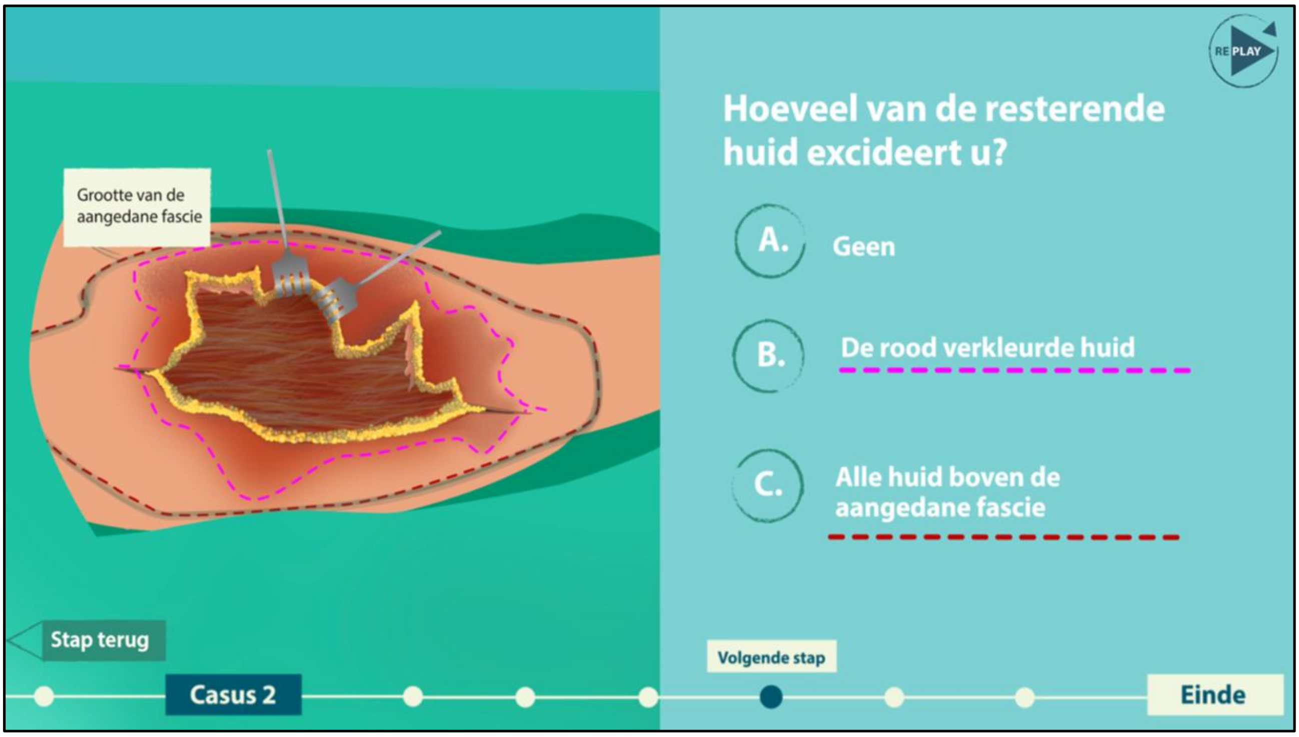

Slide 27C (if answered A to question on slide 24C)
Question right upper corner: How much skin do you excise?

A: None
B: The red discolored skin
C: All skin above the affected fascia

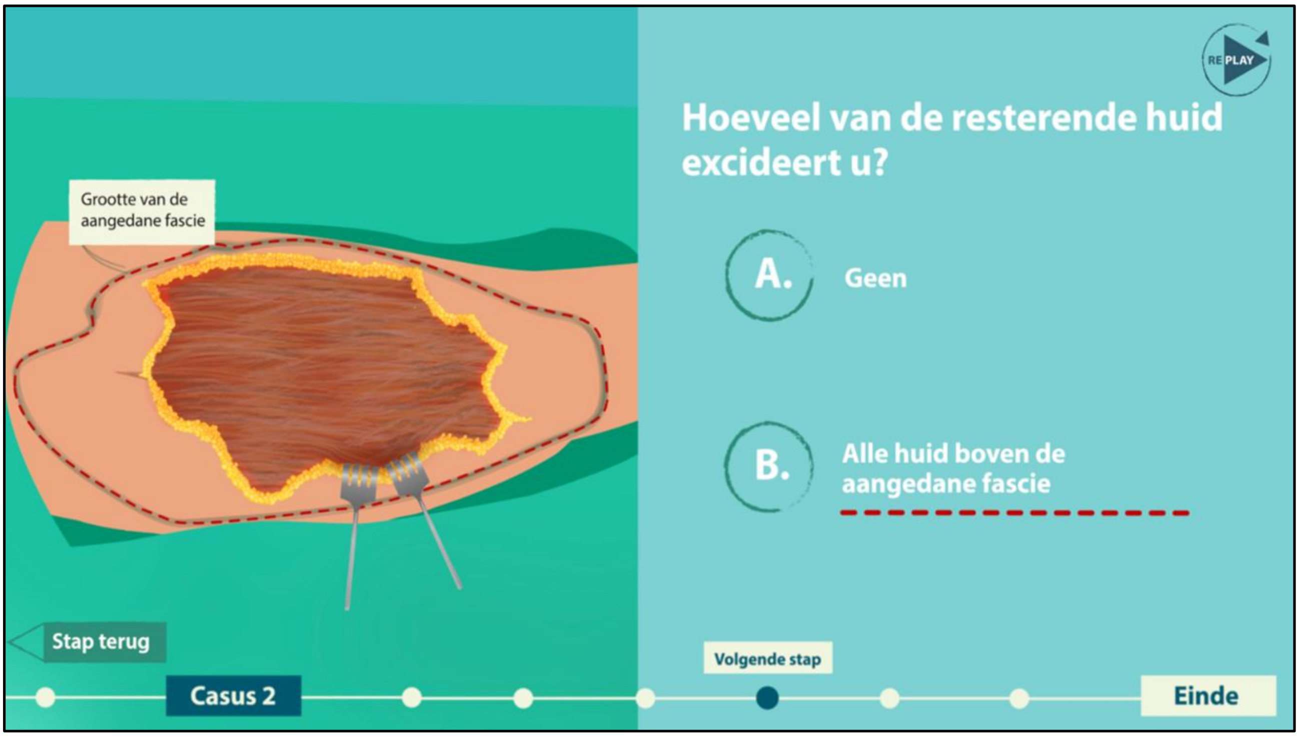

Slide 27D (if answered D to question on slide 24)
Question right upper corner: How much skin do you excise?

A: None
B: All skin above the affected fascia

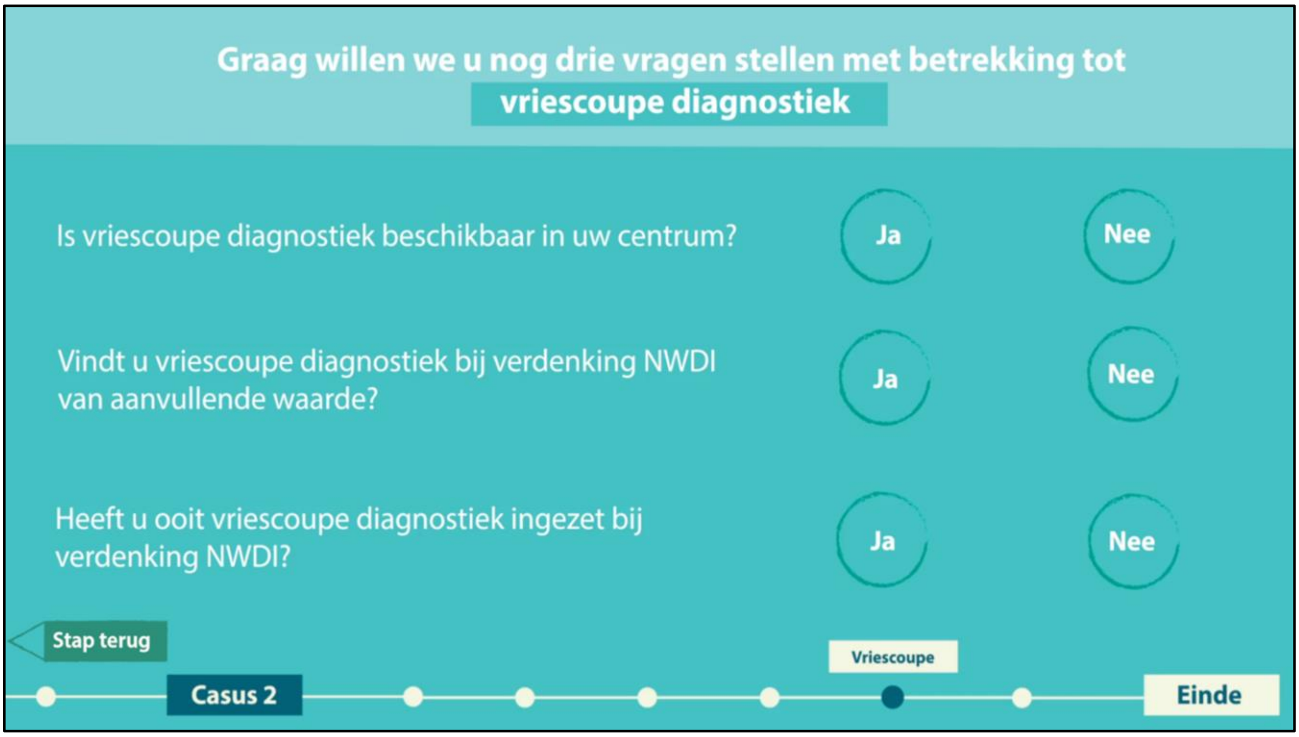

Slide 28
We would like to ask you three questions regarding frozen section diagnostics

Is frozen section diagnostics available in your hospital? Yes/No
Do you think that frozen section diagnostics is of additional value by suspicion of NSTI? Yes/No
Did you ever use frozen section diagnostics by suspicion of NSTI? Yes/No

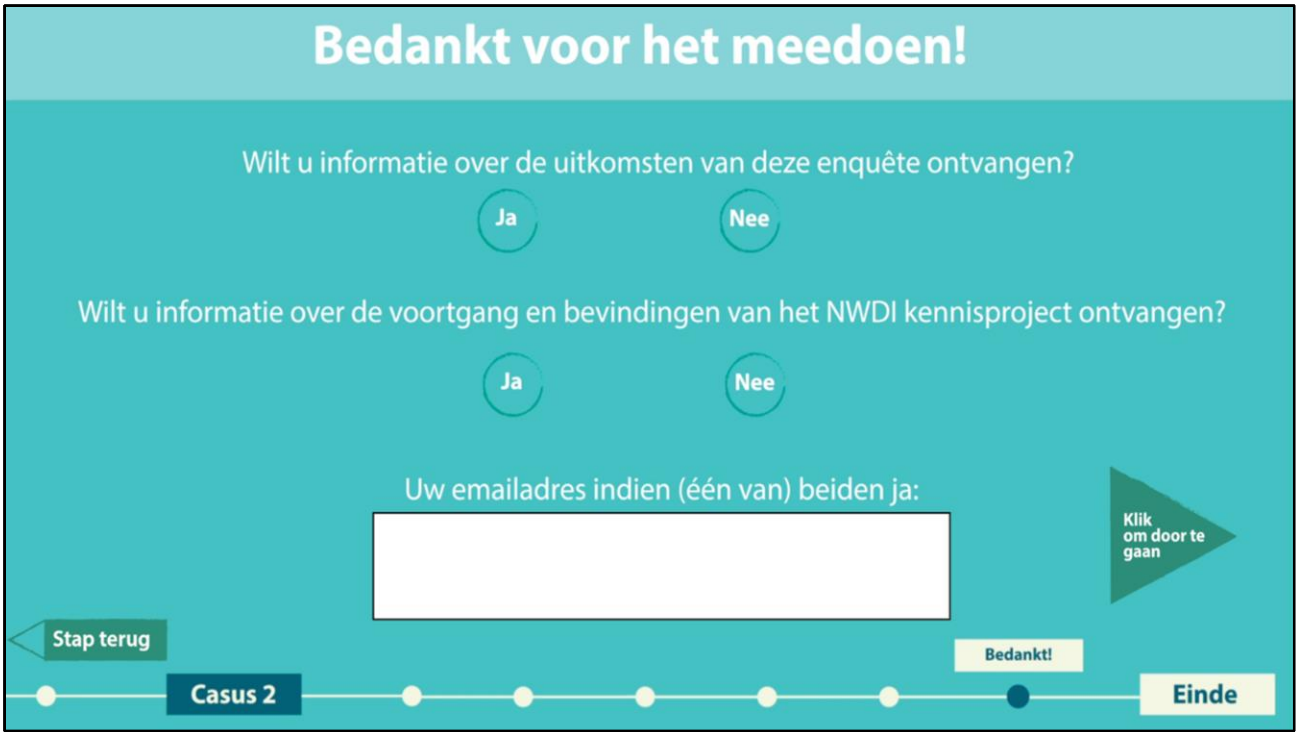

Slide 29
Thank you for participating!

Would you like to receive the results from this survey? Yes/No
Would you like to receive information about the progress and findings of the NSTI Knowledge Project? Yes/No

Your e-mailadress if (one of) both is yes:

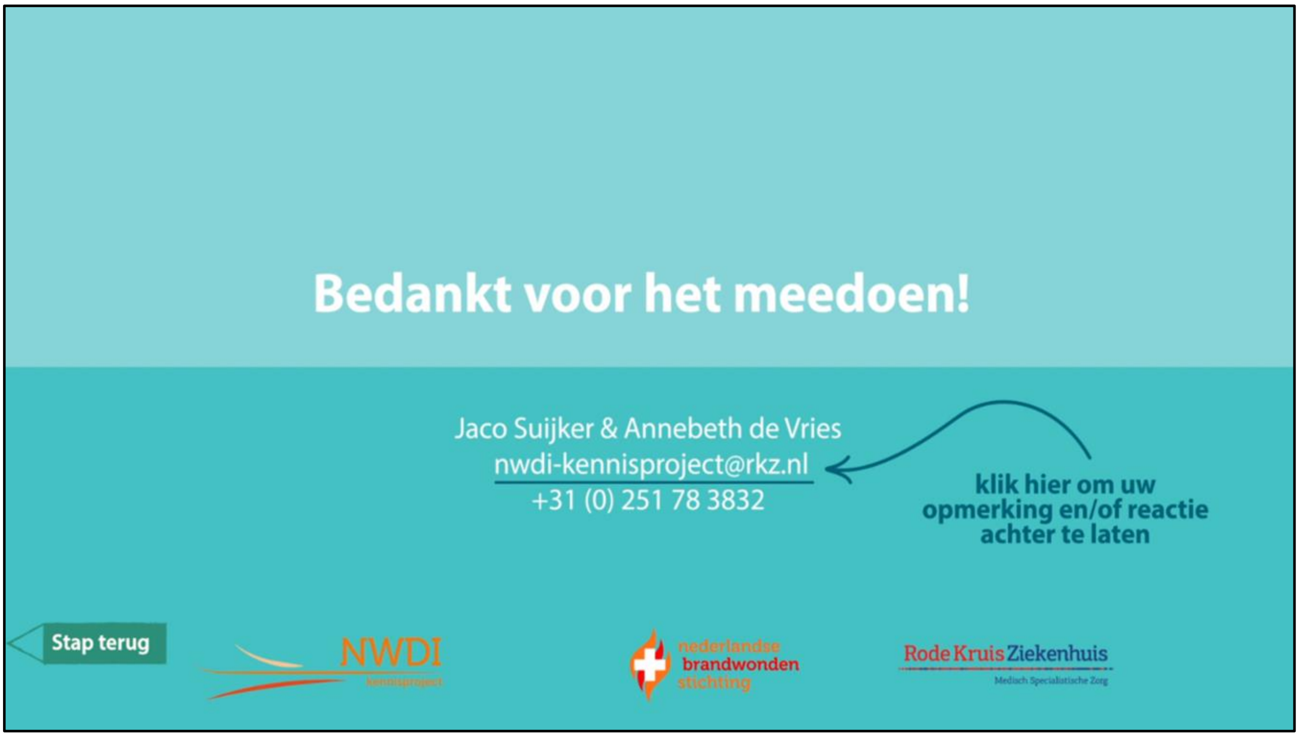

Slide 30
Thank you for participating!

Blue tekst: Klick here to leave comments or a response

The next slides concern the additional questions sent to respondents who self willingly filled in their e-mail adress at the end of the survey.

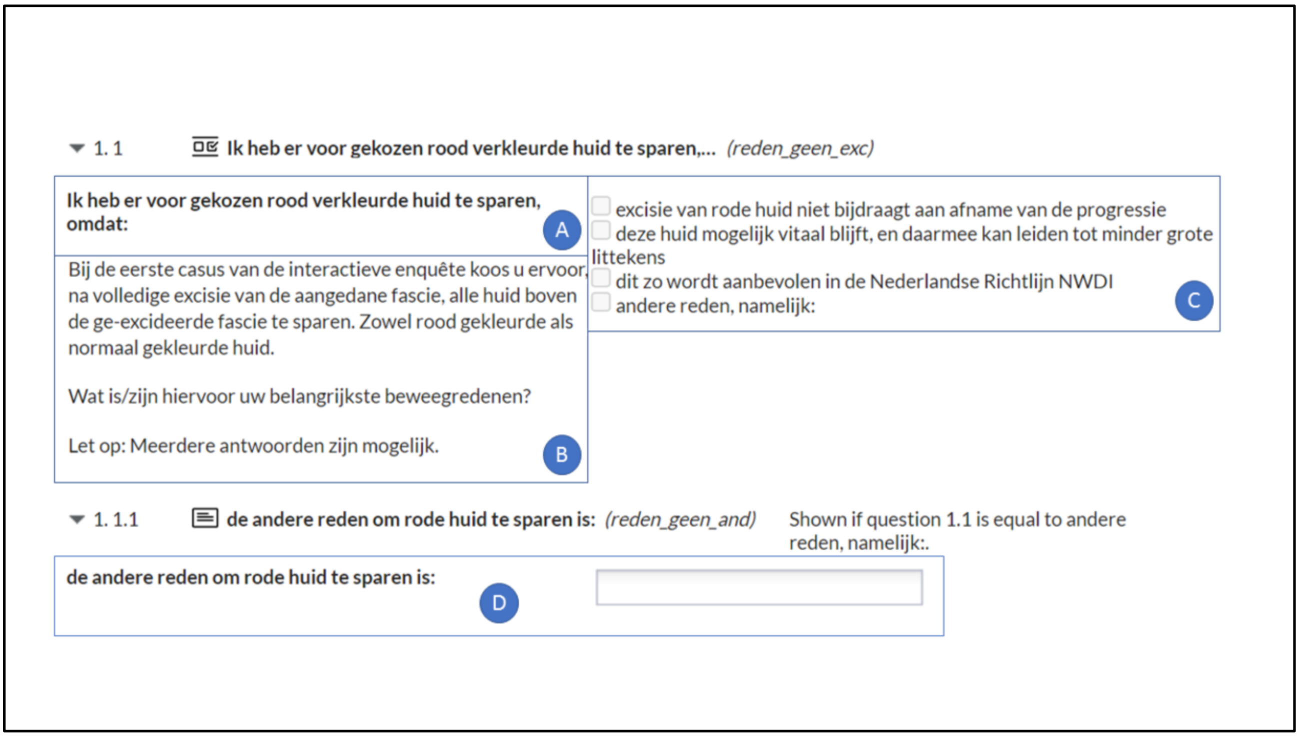

1. 1 Ik heb er voor gekozen rood verkleurde huid te sparen,... *(reden_geen_exc)*

**Ik heb er voor gekozen rood verkleurde huid te sparen, omdat:** (A)	excisie van rode huid niet bijdraagt aan afname van de progressie<br>deze huid mogelijk vitaal blijft, en daarmee kan leiden tot minder grote littekens<br>dit zo wordt aanbevolen in de Nederlandse Richtlijn NWDI<br>andere reden, namelijk: (C)
Bij de eerste casus van de interactieve enquête koos u ervoor, na volledige excisie van de aangedane fascie, alle huid boven de ge-excideerde fascie te sparen. Zowel rood gekleurde als normaal gekleurde huid.<br><br>Wat is/zijn hiervoor uw belangrijkste beweegredenen?<br><br>Let op: Meerdere antwoorden zijn mogelijk. (B)	

1. 1.1 de andere reden om rode huid te sparen is: *(reden_geen_and)* Shown if question 1.1 is equal to andere reden, namelijk:.

de andere reden om rode huid te sparen is: (D)

A:
I chose to preserve red colored skin:

B:
In case 1 of the interactive survey you chose, after complete excision of the affected fascia, to preserve all skin above the excised fascia. Red colored as well as normal colored skin.

What is/are the most important motives for u to do so?

Pay attention: multiple answers are possible

C:
1. Excision of the red skin does not contribute to a decrease in progression
2. This skin might stay vital, and because of that can lead to less extensive scars
3. It is recommended like this in the Dutch guideline NSTI
4. Other reason, namely:

D:
The other reason to preserve red skin is:

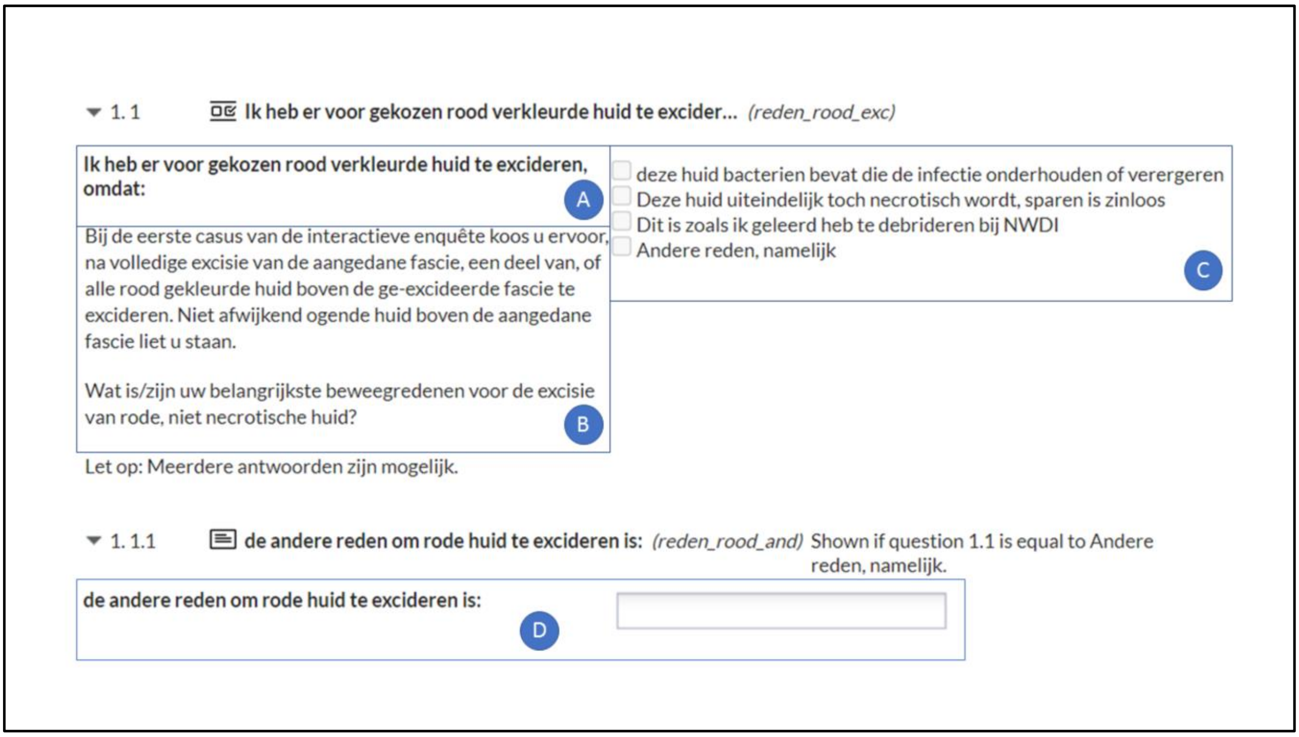

1.1 Ik heb er voor gekozen rood verkleurde huid te excider... *(reden_rood_exc)*

**Ik heb er voor gekozen rood verkleurde huid te excideren, omdat:** (A)	☐ deze huid bacterien bevat die de infectie onderhouden of verergeren<br>☐ Deze huid uiteindelijk toch necrotisch wordt, sparen is zinloos<br>☐ Dit is zoals ik geleerd heb te debrideren bij NWDI<br>☐ Andere reden, namelijk (C)
Bij de eerste casus van de interactieve enquête koos u ervoor, na volledige excisie van de aangedane fascie, een deel van, of alle rood gekleurde huid boven de ge-excideerde fascie te excideren. Niet afwijkend ogende huid boven de aangedane fascie liet u staan.<br><br>Wat is/zijn uw belangrijkste beweegredenen voor de excisie van rode, niet necrotische huid? (B)	

Let op: Meerdere antwoorden zijn mogelijk.

1.1.1 **de andere reden om rode huid te excideren is:** *(reden_rood_and)* Shown if question 1.1 is equal to Andere reden, namelijk.

**de andere reden om rode huid te excideren is:** (D)	

A:
I chose to excise red coloured skin because:

B:
In case one of the interactive survey you chose, after complete excision of the affected fascia, to excise all red and normal colored skin over the excised fascia.

What is/are the most important motives for u to excise the red skin?

Pay attention: multiple answers are possible

C:
1. This skin contains bacteria that maintain or worsen the infection
2. This skin will eventually turn necrotic, preserving it is pointless
3. This is the way I learned to debride in case of NSTI
4. Other reason, namely:

D:
The other reason to excise red skin is:

▾ 2.1 Ik heb er voor gekozen niet verkleurde huid te excider... *(reden_norm_exc)*

Ik heb er voor gekozen niet verkleurde huid te excideren, omdat: (A)

Bij de eerste casus van de interactieve enquête koos u ervoor, na volledige excisie van de aangedane fascie, alle huid boven de aangedane fascie te excideren.

Wat is/zijn uw belangrijkste beweegredenen voor de excisie van niet verkleurde, normaal ogende huid?

Let op: Meerdere antwoorden zijn mogelijk (B)

- ☐ deze huid bacterien bevat die de infectie onderhouden of verergeren
- ☐ Deze huid uiteindelijk toch necrotisch wordt, sparen is zinloos
- ☐ Dit is zoals ik geleerd heb te debrideren bij NWDI
- ☐ Andere reden, namelijk

(C)

▾ 2.1.1 **de andere reden om niet verkleurde huid te sparen is:** *(reden_norm_oth)* — Shown if question 2.1 is equal to Andere reden, namelijk.

de andere reden om niet verkleurde huid te sparen is: (D)

A:
I chose to excise non discolored skin because:

B:
In case one of the interactive survey you chose, after complete excision of the affected fascia, to excise all red and normal coloured skin over the excised fascia.

What is/are the most important motives for u to excise the normal colored skin?

Pay attention: multiple answers are possible

C:
1. This skin contains bacteria that maintain or worsen the infection
2. This skin will eventually turn necrotic, preserving it is pointless
3. This is the way I learned to debride in case of NSTI
4. Other reason, namely:

D:
The other reason to excise non discolored skin is:

Appendix B. Complement to Material and Methods.

Response rate estimation

For general surgeons, a direct mailing was not possible, due to restrictions of the Dutch Association for Surgery. Those members received a link in the monthly newsletter, which linked to an invitation on the website of the Dutch Association for Surgery. Since only 164 general surgeons clicked on the hyperlink in the newsletter, complementary recruitment strategies were initiated. First, 70 WhatsApp messages were sent to general surgeons (contacts of AMV) two weeks after the mailing, followed by emails to 43 heads of surgical education centers throughout the Netherlands. Both WhatsApp and e-mails invitations requested to personally participate and spread the link among other surgeons. To estimate the response rate for these 113 surgeons contacted by WhatsApp or email, it was assumed that each of the approached surgeons would contact three colleagues on average. This results in an estimated number of surgeons reached by these complementary recruitment strategies of 452. Combined with those reached by the link, the total number of surgeons reached was expected to be 616.

Data transposition and regression

For analysis purposes (correlation and regression analysis), answers for both cases were transposed to an ordinal scale of 1-4; ranging from the approach in which most potentially viable skin was preserved (4), to resecting all skin, including normal looking skin, above the affected fascia (1). In case 2, which had five answer options, only four were part of the scale; option A was excluded since it represented a generally accepted incorrect answer, which was to preserve evidently necrotic skin.

Correlation between cases was assessed using spearman's Rs test and reported as correlation coefficient. Ordinal logistic regression was used to identify predictors of surgical

approach. Outcomes were reported as estimated mean differences and 95% confidence intervals (CIs).

Preventing multiple entries from the same individual

In order to prevent multiple entries from the same participant, a log file was followed closely. Surveys initiated shortly after each other containing the exact same characteristics of the participant were removed by the head investigator when the IP address was identical. In that case the latest completed survey was saved.

Survey administration

Data were collected automatically from the survey in the MongoDB database as Binary JavaScript Object Notation (BSON). It was sent to a secured file that will exist for five years and is only accessible to the head investigator and a local research coordinator. Each week the collected data was shared with the research team, to track progression and take appropriate steps if needed.
